# Comparative genomics reveals the correlations of stress response genes and bacteriophages in developing antibiotic resistance of *Staphylococcus saprophyticus*

**DOI:** 10.1128/msystems.00697-23

**Published:** 2023-12-05

**Authors:** Kailun Zhang, Robert F. Potter, Jamie Marino, Carol E. Muenks, Matthew G. Lammers, Jennifer Dien Bard, Tanis C. Dingle, Romney Humphries, Lars F. Westblade, Carey-Ann D. Burnham, Gautam Dantas

**Affiliations:** 1 Department of Pathology and Immunology, Division of Laboratory and Genomic Medicine, Washington University School of Medicine in St. Louis, St. Louis, Missouri, USA; 2 The Edison Family Center for Genome Sciences and Systems Biology, Washington University School of Medicine in St. Louis, St. Louis, Missouri, USA; 3 Department of Pathology and Laboratory Medicine, Weill Cornell Medicine, New York, USA; 4 Department of Pathology and Laboratory Medicine, Children’s Hospital Los Angeles, Los Angeles, California, USA; 5 Keck School of Medicine, University of Southern California, Los Angeles, California, USA; 6 Department of Pathology and Laboratory Medicine, University of Calgary, Calgary, Alberta, Canada; 7 Department of Pathology, Microbiology, and Immunology, Vanderbilt University School of Medicine, Nashville, Tennessee, USA; 8 Department of Medicine, Washington University School of Medicine in St. Louis, St. Louis, Missouri, USA; 9 Department of Molecular Microbiology, Washington University School of Medicine in St. Louis, St. Louis, Missouri, USA; 10 Department of Pediatrics, Washington University School of Medicine in St. Louis, St. Louis, Missouri, USA; 11 Department of Biomedical Engineering, Washington University in St. Louis, St. Louis, Missouri, USA; University of Illinois, Chicago, Illinois, USA

**Keywords:** *Staphylococcus saprophyticus*, AMR, phage-carrying ARG

## Abstract

*Staphylococcus saprophyticus* is the leading Gram-positive cause of uncomplicated urinary tract infections. Recent reports of increasing antimicrobial resistance (AMR) in *S. saprophyticus* warrant investigation of its understudied resistance patterns. Here, we characterized a diverse collection of *S. saprophyticus* (*n* = 275) using comparative whole genome sequencing. We performed a phylogenetic analysis of core genes (1,646) to group our *S. saprophyticus* and investigated the distributions of antibiotic resistance genes (ARGs). *S. saprophyticus* isolates belonged to two previously characterized lineages, and 14.91% (41/275) demonstrated multidrug resistance. We compared antimicrobial susceptibility phenotypes of our *S. saprophyticus* with the presence of different ARGs and gene alleles. 29.8% (82/275) carried staphylococcal cassette chromosome mobile elements, among which 25.6% (21/82) were *mecA*^+^. Penicillin resistance was associated with the presence of *mecA* or *blaZ*. The *mecA* gene could serve as a marker to infer cefoxitin and oxacillin resistance of *S. saprophyticus*, but the absence of this gene is not predictive of susceptibility. Utilizing computational modeling, we found several genes were associated with cefoxitin and oxacillin resistance in *mecA^−^* isolates, some of which have predicted functions in stress response and cell wall synthesis. Furthermore, phenotype association analysis indicates ARGs against non-β-lactams reported in other staphylococci may serve as resistance determinants of *S. saprophyticus*. Lastly, we observed that two ARGs [*erm* and *erm (44)v*], carried by bacteriophages, were correlated with high phenotypic non-susceptibility against erythromycin (11/11 and 10/10) and clindamycin (11/11 and 10/10). The AMR-correlated genetic elements identified in this work can help to refine resistance prediction of *S. saprophyticus* during antibiotic treatment.

**IMPORTANCE:**

*Staphylococcus saprophyticus* is the second most common bacteria associated with urinary tract infections (UTIs) in women. The antimicrobial treatment regimen for uncomplicated UTI is normally nitrofurantoin, trimethoprim-sulfamethoxazole (TMP-SMX), or a fluoroquinolone without routine susceptibility testing of *S. saprophyticus* recovered from urine specimens. However, TMP-SMX-resistant *S. saprophyticus* has been detected recently in UTI patients, as well as in our cohort. Herein, we investigated the understudied resistance patterns of this pathogenic species by linking genomic antibiotic resistance gene (ARG) content to susceptibility phenotypes. We describe ARG associations with known and novel SCC*mec* configurations as well as phage elements in *S. saprophyticus*, which may serve as intervention or diagnostic targets to limit resistance transmission. Our analyses yielded a comprehensive database of phenotypic data associated with the ARG sequence in clinical *S. saprophyticus* isolates, which will be crucial for resistance surveillance and prediction to enable precise diagnosis and effective treatment of *S. saprophyticus* UTIs.

## INTRODUCTION

In the 1960s, a few coagulase-negative staphylococci (CoNS) strains were isolated from the urine of women with acute urinary tract infection (UTI) (1), which were later classified as *Staphylococcus saprophyticus*. To date, *S. saprophyticus* has been reported as the second most frequent causative organism of uncomplicated UTIs in women (2). Infrequently, it is also responsible for several complications including acute pyelonephritis (3, 4), bloodstream infection (5), endocarditis (6), and nephrolithiasis (7). In terms of body habitats, *S. saprophyticus* is present in humans as part of the normal microbiota of the skin and mucosal surfaces in perineum, rectum, urethra, cervix, and gastrointestinal tract (2, 8). Studies have shown that rectal, urethral, and vaginal colonization of *S. saprophyticus* is associated with UTIs caused by this organism (9, 10). This species is also widely distributed in the environment (11, 12). Antimicrobial selection for treatment of patients with uncomplicated *S. saprophyticus* UTIs is typically performed without *in vitro* antibiotic susceptibility testing (AST) data, as suggested by current Clinical Laboratory Standards Institute (CLSI) guidelines (13): isolates of this species are typically considered to be susceptible to antimicrobials commonly used to treat UTI; thus, routine AST for isolates from the urinary tract is not advised; the antibiotics of choice are typically nitrofurantoin and trimethoprim-sulfamethoxazole (TMP-SMX). However, two recent studies noted that 17.9% and 9.0% of their UTI *S. saprophyticus* isolates from Brazil and Iran, respectively, were resistant to TMP-SMX (14, 15). This highlights the importance of considering specific local resistance patterns when choosing appropriate antibiotic coverage during UTI treatment.

Whole genome sequencing (WGS) of bacterial strains has become a desired method for profiling resistance determinants (referred to as the “genotype”) of various pathogens. Linking genomic antibiotic resistance gene (ARG) content to antibiotic resistance phenotypes is crucial for resistance surveillance. This has been recently assessed for *Escherichia coli* (16–18), *Klebsiella pneumoniae* (16, 19), *Mycobacterium tuberculosis* (20, 21), *Neisseria gonorrhoeae* (22, 23), *Pseudomonas aeruginosa* (24, 25), *Shigella sonnei* (26), and *Staphylococcus aureus* (27–29). A recent WGS analysis of *S. saprophyticus* from human UTIs and the pig-meat processing chain reported that they belonged to two major lineages, G and S, and identified a strong association between ARGs, phages, platelet-binding proteins (PBPs), and an increased genomic recombination rate (30). These studies and the growing reports of increasing phenotypic resistance in *S. saprophyticus* prompt a comprehensive investigation of resistome genotype to phenotype associations for *S. saprophyticus*.

Here, we performed comparative WGS on a global collection of 275 clinical *S. saprophyticus* isolates and compared their resistome genotypic profiles with their phenotypic susceptibilities. Consistent with previous reports (30), our *S. saprophyticus* strains also separate into two major lineages based on their core gene phylogenetic identities. We observed different ARG patterns and distributions between lineages. To identify genomic determinants of resistance, we first examined the correlations between susceptibility phenotypes and ARGs that had well-known relationships to resistance in staphylococci, e.g., *mecA* for β-lactam resistance (31) and *tet(K*) for doxycycline resistance (32, 33). Then, we utilized computational modeling to identify novel genes or mutations that were significantly associated with key resistance phenotypes of *S. saprophyticus*. Finally, given the potential role of bacteriophages in ARG transmission (34–37), we detected ARGs within phage elements in our cohort and found that they were associated with high phenotypic resistance against erythromycin/clindamycin antibiotics.

## RESULTS

### Lineages of *S. saprophyticus* isolates exhibit different resistance patterns

**Fig 1:**
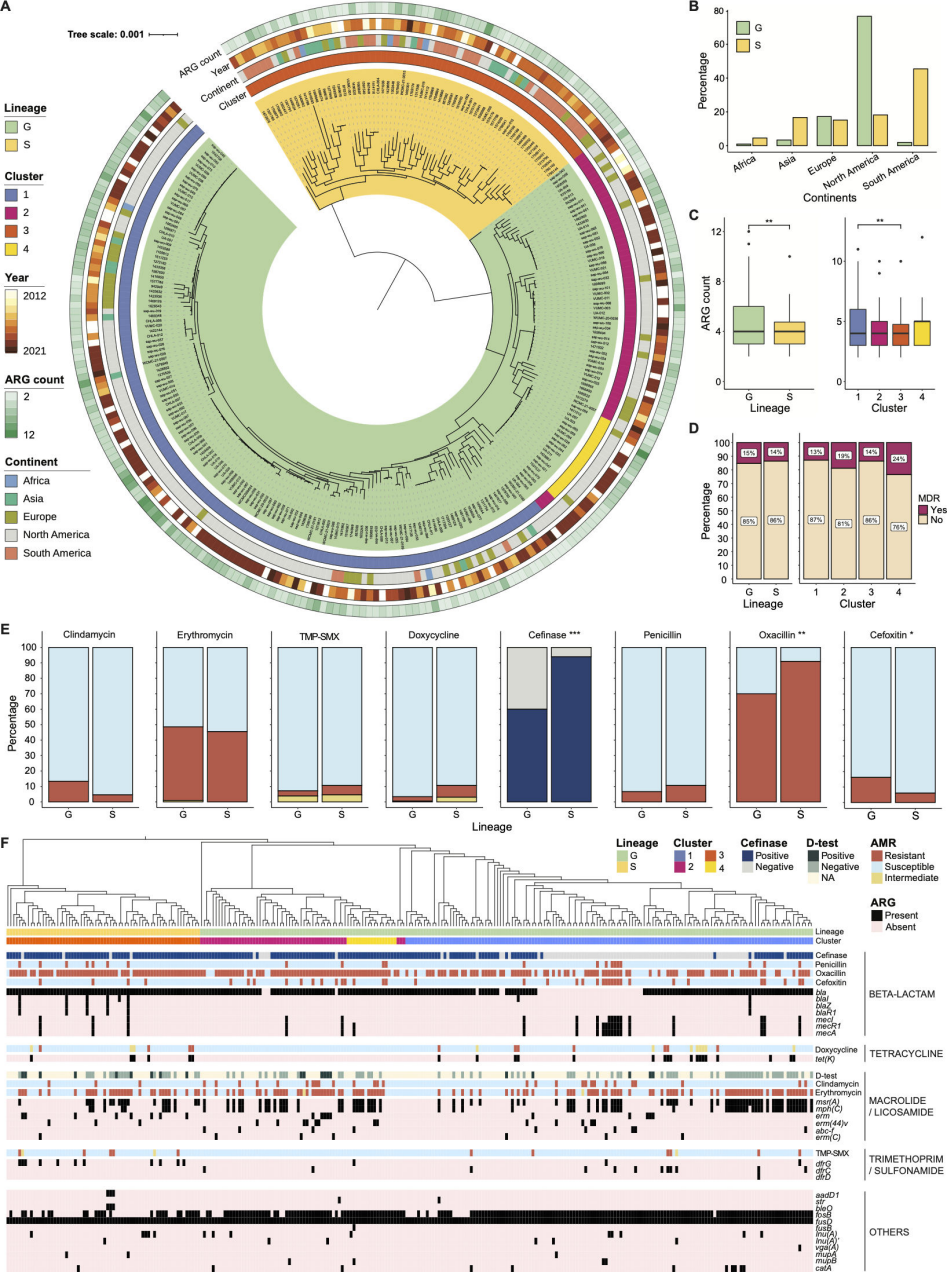
Relatedness and antibiotic resistance profile of *S. saprophyticus* recovered globally from human infections and colonization during 2012–2021. (**A**) Phylogenetic tree demonstrating the core gene alignment of 275 *S*. *saprophyticus* isolates. Each node represents an isolate. Two lineages are indicated with color ranges covering the complete clade branches. Hierarchical clusters based on rhierBAPS are indicated by color strips in the internal ring. The continent of origin and year of collection, as well as the number of ARGs carried by each isolate, are labeled by color strips. The scale bar represents the average number of nucleotide substitutions. (**B**) Distribution of *S. saprophyticus* isolates in terms of their continents in the two lineages. (**C**) Box plot comparing the number of ARG between isolates from different lineages (left) and clusters (right). (**D**) Comparing multidrug resistance (MDR) rates between isolates from different lineages (left) and clusters (right). (**E**) Comparing percentages of isolates resistant to different antibiotics or expressing β-lactamase activity between lineages. Susceptibility phenotypes: resistant, orange; intermediate, yellow; susceptible, light blue. β-lactamase activity: positive, dark blue; negative, light gray. In D and E, the χ^2^ test is used with a significance threshold of 0.05. * *P* < 0.05, **P < 0.01, and ***P < 0.001. (**F**) Annotating the core gene phylogenetic tree with antibiotic resistance phenotypes, linking with the presence of various ARGs. Genotypical and phenotypical data are grouped by antimicrobial class. Lineages, clusters, resistance phenotypes, and ARG content are indicated by color strips.

We compared the annotated WGS of our *S. saprophyticus* isolates (*n* = 275) and found that the total pan-genome included 9,584 genes. Among these, 1,646 were considered core genes (>99% prevalence), 1,421 were considered shell genes (15%–99% prevalence), and 6,517 were cloud genes (<15% prevalence). We used core gene alignments to build a maximum-likelihood phylogenetic tree (Fig. 1A; Table S1). The “water striders” shape (38) of our *S. saprophyticus* tree exhibited a long internal branch separating two very distinct sub-populations (Fig. S1A). This result was consistent with a prior report of *S. saprophyticus*, though their genomic phylogeny was built on whole-genome single nucleotide polymorphisms (SNPs) mapped to a single reference *S. saprophyticus* strain ATCC 15305, and they designated the two subpopulations as lineage G and S (30). To confirm that lineage definitions were not biased by sampling, as well as to assign the lineage group of our isolates, we generated a combined core gene alignment of all published *S. saprophyticus* genomes and the genomes from our work (Fig. S1B). We confirmed that these genomes were separated into two major groups, and all G and S isolates from Lawal et al. belonged to different groups. Thus, we proceeded with utilizing G and S as the lineage names in our study. Among our cohort, 76% (209/275) of *S. saprophyticus* isolates were from lineage G, which differed by between 16 and 4,429 core gene single nucleotide polymorphisms (cgSNPs) with a whole-genome average nucleotide identity (ANI) of 99.2116%–99.9971%. Our isolates from lineage S (*n* = 66) had cgSNPs of 0–5,182 with an ANI of 99.2776%–99.9997% (Fig. S1C). The cgSNP distances and whole genome ANI compared between G and S isolates were 8,720–10,967 and 98.5267%–99.1449%, respectively (Fig. S1C). Isolates from lineage G were mostly from North America (160/209), while lineage S had more isolates collected from South America (30/66; Fig. 1B). Intriguingly, the tree shapes of the two lineages are dissimilar, indicating potentially distinct evolutionary patterns. To confirm this assumption, we utilized rhierBAPS (39, 40) to hierarchically cluster the core genes of *S. saprophyticus*. Four clusters were detected at level 1, among which three were from lineage G (cluster 1, *n* = 139; cluster 2, *n* = 53; cluster 4, *n* = 17), and all S isolates were characterized as one cluster (cluster 3; Fig. 1A). Furthermore, using principle coordinates analysis (PCoA) on the presence-absence matrix representing all accessary (i.e., non-core) genes, we found that different lineages or clusters mixed within the plot, indicating that *S. saprophyticus* accessory gene content does not recapitulate the core gene structure (Fig. S1D).

Next, we identified ARGs encoded by *S. saprophyticus* and compared their distributions between lineages or clusters. We detected 29 ARGs of 9 antimicrobial categories in our cohort. The antimicrobial categories were used to describe the acquired resistance profile in *S. aureus* (41). All *S. saprophyticus* isolates carried at least two ARGs, and one isolate (UA-007) had up to 12 ARGs (Fig. 1A). The numbers of ARGs of G isolates (range: 2–12) were significantly larger than those in S isolates (range: 2–10) determined by Wilcoxon rank-sum test (*P*-value is 0.0016), although both contained a median of four ARGs (Fig. 1C). When comparing among clusters, we only observed differences in ARG numbers carried by isolates from cluster 1 and 3 (*P*-value is 0.0012 by Wilcoxon rank-sum test; Fig. 1C). We determined antimicrobial resistance (AMR) phenotypes and the β-lactamase activities of our *S. saprophyticus* isolates by disk diffusion and Cefinase assays, respectively. 14.91% (41/275) *S*. *saprophyticus* isolates demonstrated multidrug resistance (MDR), defined as the isolate was non-susceptible to at least one agent in more than three antimicrobial categories (41), including β-lactams, folate pathway inhibitor, lincosamides, macrolides, and tetracyclines. Specifically, 7/41 isolates were MDR due to their non-susceptibility against β-lactams, macrolides, and tetracyclines; 24/41 isolates were MDR due to their non-susceptibility against β-lactams, lincosamides, and macrolides; 8/41 isolates were MDR due to their non-susceptibility against β-lactams, folate pathway inhibitor, and macrolides; 1/41 isolate was MDR due to its non-susceptibility against β-lactams, folate pathway inhibitor, macrolides, and tetracyclines; and 1/41 isolate was MDR due to its non-susceptibility against β-lactams, folate pathway inhibitor, lincosamides, and macrolides. We observed no differences in MDR rates between lineages or clusters (χ^2^ test, *P*-values are 0.74 and 0.54, respectively; Fig. 1D). Furthermore, the β-lactamase activity and the resistance rates (the number of resistant isolates to non-resistant isolates) against cefoxitin and oxacillin were significantly different between the two lineages (χ^2^ test, *P*-value is 0.0005, 0.0435, and 0.0015; Fig. 1E); the β-lactamase activity and the resistance rates against clindamycin, doxycycline, TMP-SMX, cefoxitin, and oxacillin were significantly different between clusters (Fig. S1E). To visualize the AMR patterns of each individual isolate, we grouped genetic data with susceptibility phenotypes in the core gene phylogenetic tree (Fig. 1F; Table S2). In sum, our globally diverse collection of human pathogenic *S. saprophyticus* comprises two major lineages that exhibit distinct AMR burdens. Both genotypical and phenotypical data indicate the development of MDR in our cohort.

### Non-typeable SCC elements were identified in *S. saprophyticus* linked with AMR

**Fig 2:**
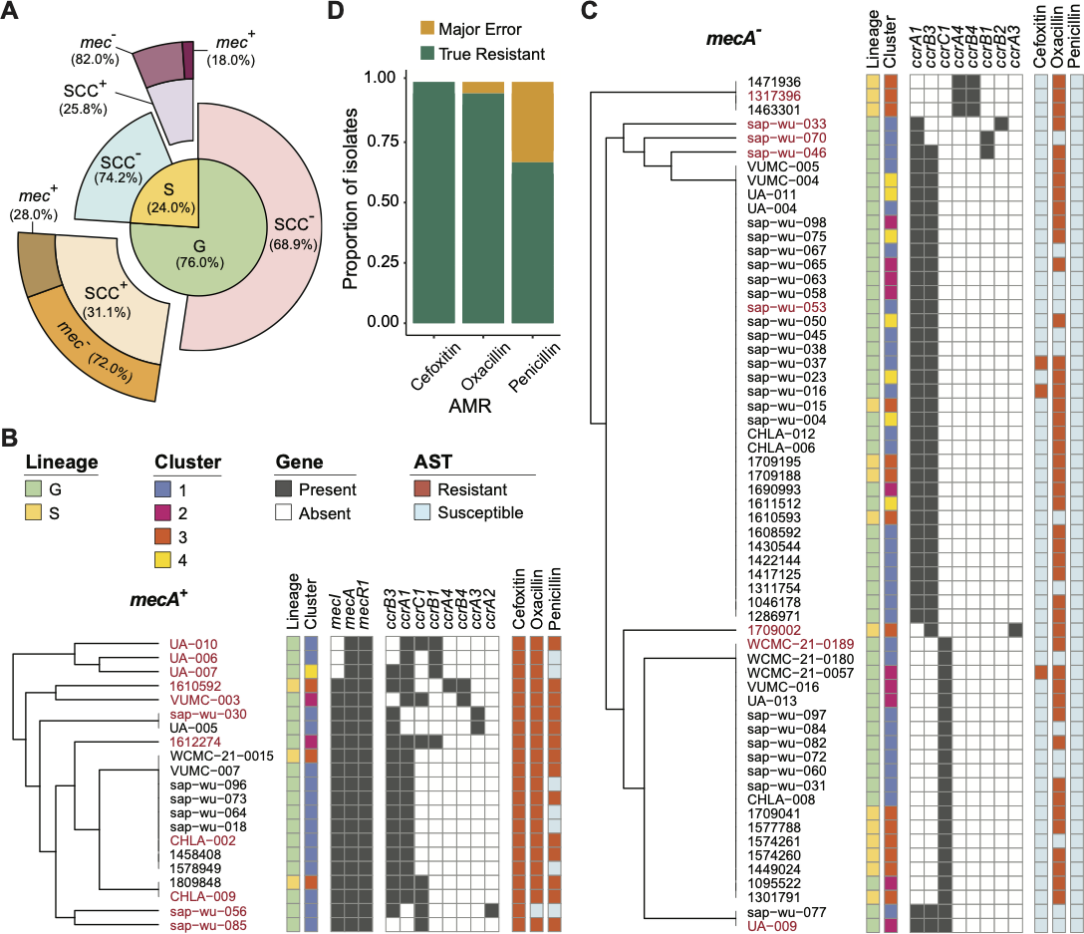
Diversity of SCC elements in *S. saprophyticus* and their resistances against β-lactams. (**A**) Distribution of SCC elements in G and S lineages. (**B**) Hierarchical tree of 21 isolates with SCC*mec* structured by the composition of *mec* and *ccr* genes. (**C**) Hierarchical tree of 61 isolates with SCC elements but not carrying *mecA*, structured by the composition of *ccr* genes. Lineages, clusters, and resistance phenotypes are indicated by color strips in B and C. (**D**) Prediction accuracy of genotype to phenotype inference for the 21 *mecA*^+^ strains of the study. It is used *mecA* gene as the marker to predict the resistance against three β-lactam antibiotic drugs.

Staphylococcal cassette chromosome *mec* (SCC*mec*) is a genetic mobile element that conveys the central determinant of the broad-spectrum β-lactam resistance encoded by the *mecA* gene (42). Additionally, SCC*mec* element often carries site-specific recombinases designated as cassette chromosome recombinases (*ccr*) (43, 44). To date, 11 SCC*mec* types have been characterized for *S. aureus* based on the compositions of their *mec* and *ccr* genes (45), and SCC elements that do not carry *mec* gene have also been observed (46). Given the roles of SCC elements in transmitting methicillin resistance, we assessed the distribution of SCC elements in *S. saprophyticus*. Among our isolates, 29.8% (82/275) carried *ccr* genes, and the incidence of *mecA* gene was 7.6% (21/275; Fig. 2A). There was no difference in SCC*mec* prevalence between lineages (Fig. S2A). Within the SCC^+^ isolates, *rlmH* was located at the 5’-end of the SCC element (Fig. S2B and C), suggesting the insertion site of SCC element was overlapping with *rlmH*, a similar organization to the ones in other staphylococcal species (47). Gene *rlmH*, encoding rRNA large subunit methyltransferase H, was detected in all (275/275) *S. saprophyticus* by reciprocal BLAST, signifying their role to serve as recipients of SCC transferring. Additionally, SCC elements in *S. saprophyticus* contained other characteristic genes which have been reported in methicillin-resistant *S. aureus* (MRSA) (48), such as capsule gene cluster (*cap*), copper resistance (*cop*), cadmium resistance (*cad*), or the arsenic resistance operon (*ars*; Fig. S2B and C).

We attempted to determine SCC types (45) in *S. saprophyticus* based on the gene structure of *ccr* and *mec* complexes (Fig. 2B and C). We found that 3/21 of the *mec* complexes found in our cohort belonged to class B [composed of *mecA*, a truncated *mecR1* resulting from the insertion sequence IS1272 upstream of *mecA*, and IS431 downstream of *mecA* (45)] and 16/21 belonged to class A [contains *mecA*, the complete *mecR1* and *mecI* regulatory genes upstream of *mecA*, and IS431 downstream of *mecA* (45)]. IS431 sequences were detected in most SCC*mec*-positive isolates but on separated contigs with *mec* or *ccr* genes. Except for that, two isolates (1809848 and CHLA-009) showed the coexistence of *mec* genes and IS256 in their genomes (Fig. S2B). On the other hand, the compositions of *ccr* complexes of *S. saprophyticus* were more diverse and novel compared to those in MRSA. Specifically, 8/21 *mecA*^+^ SCC*mec* and 2/61 *mecA^−^* SCC elements were identified as carrying two *ccr* gene complexes. The most common *ccr* combination was *ccrA1/ccrB3* (*n* = 48), and others included *ccrA1/ccrB1* (*n* = 4), *ccrA1/ccrB2* (*n* = 1), *ccrA1/ccrB4* (*n* = 1), *ccrA2/ccrB3* (*n* = 1), *ccrA3/ccrB3* (*n* = 3), and *ccrA4/ccrB4* (*n* = 4). Among these, only *ccrA1/ccrB1*, *ccrA3/ccrB3*, and *ccrA4/ccrB4* were reported in MRSA. In addition, gene *ccrC1* was detected in 28 isolates. Two isolates (1612274 and sap-wu-046) carried *ccrA1* and a new allele of *ccrB* united *ccrB1* (1–875nt) and *ccrB3* (940–1,626 nt; Fig. S2B and C). In summary, novel SCC elements in *S. saprophyticus*, such as the ones carrying new *ccr* compositions, are non-typeable according to the current SCC*mec* classification from *S. aureus* (45). This highlights key differences among staphylococcal species and motivates further studies of the transmission of SCC elements.

In terms of AMR, we found that the *mecA* gene was able to serve as a marker to infer β-lactam resistance of *S. saprophyticus*, especially for cefoxitin and oxacillin (Fig. 2D). The resistance rates of *mecA*^+^ *S. saprophyticus* were 66.7% (14/21), 100.0% (21/21), and 95.2% (20/21) against penicillin, cefoxitin, and oxacillin, respectively (Fig. 2B), vs 2.8% (7/254), 6.7% (17/254), and 73.2% (186/254) in *mecA*^−^ isolates (Fig. 2C). Of note, the presence of the *mecA* gene was also correlated with higher ARG numbers and higher phenotypic resistance against both β-lactam and non-β-lactam antibiotics which we detected in this work (Fig. S2D and E).

### *S. saprophyticus* mutants demonstrate variable resistance patterns to β-lactam antibiotics

**Fig 3:**
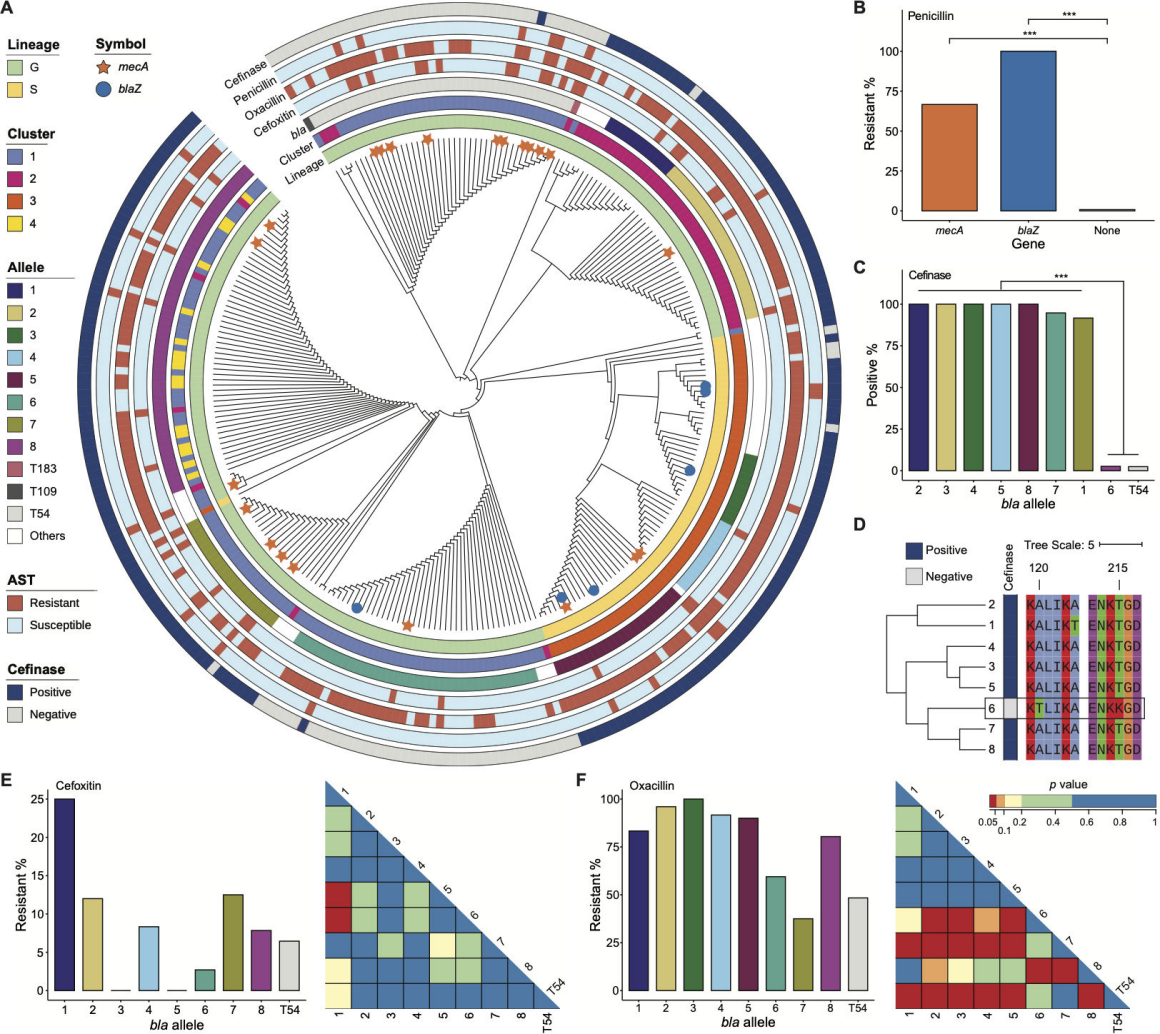
Associations between gene alleles encoding β-lactamase identified in *S. saprophyticus* and their AMR against β-lactam antibiotics. (**A**) Hierarchical tree of *bla* sequences from all *S. saprophyticus* isolates, according to amino acid sequence identity. Alleles 1–8 indicate *bla* gene alleles with at least one amino acid substitution that each is present in at least 10 isolates, otherwise are labeled as “others.” Alleles T183, T109, and T54 indicate truncated *bla* based on their putative peptide length. *S. saprophyticus* lineages, clusters, resistance phenotypes, and β-lactamase activity are indicated by color strips. The presence of *mecA* and *blaZ* genes is symbolized by the orange star and blue circle, respectively, at the tip of the branch. Tree branch length is ignored. (**B**) Percentage of isolates resistant to penicillin out of the total number of *S. saprophyticus* carrying *mecA* and *blaZ*, compared to isolates with none of them. (**C**) Percentage of isolates exhibiting β-lactamase activity, detected by Cefinase assay, out of the total number of *S. saprophyticus* carrying specific *bla* gene alleles. (**D**) Hierarchical tree of *bla* gene alleles 1–8 with the alignment of amino acid sequences. β-lactamase activity is indicated by color strips. The black box highlights the unique mutations in allele 6 with the potential to influence β-lactamase activity. The scale bar represents the average number of amino acid substitutions. See Fig. S3C for the whole-length alignment. (**E and F**) Left panel: percentage of isolates resistant to cefoxitin and oxacillin out of the total number of *mecA*^−^ *S. saprophyticus* carrying specific *bla* gene alleles. Right panel: associated *P*-value based on the χ^2^ test compared resistant rates between different alleles. The *P*-value is color-coded, and red indicates significant differences. The χ^2^ test is used with a significance threshold of 0.05 in B–C and E–F. *** *P* < 0.001.

Bacteria have developed various mechanisms to combat β-lactam antibiotics. One of the major resistance mechanisms relies on the production of β-lactamase enzymes which hydrolyze the β-lactam ring, thereby inactivating the drug (49). All our *S. saprophyticus* isolates carried a *bla* gene (class A β-lactamase, 873 bp), and six of them also encoded *blaZ* (penicillin-hydrolyzing class A β-lactamase, 846 bp; Fig. 3A). Interestingly, 83.3% (5/6) of *blaZ*^+^ isolates were from lineage S. Furthermore, penicillin resistance was higher for the isolates carrying *mecA* or *blaZ* genes (χ^2^ test, both *P*-values are 0.0005; Fig. 3A and B). Inference of penicillin resistance using *mecA* or *blaZ* as the markers showed good performances with an accuracy (true susceptible and true resistant) of 96.73% (266/275; Fig. S3A). The prediction errors (major error and very major error are 7/265 and 2/265, respectively) were from the isolates only carrying *mecA* but not *blaZ*, although no relationship was found between *mecA* variants and penicillin phenotypes (Fig. S3B).

Next, we generated a hierarchical tree based on the amino acid sequence identities of *bla* (Fig. 3A) and tested the associations of different mutations with β-lactamase activities and β-lactam resistance. We defined different *bla* alleles if they carried a single amino acid substitution; alleles were numbered and analyzed if they were present in at least 10 isolates, otherwise were labeled as “Others”; T183, T109, and T54 represented truncated *bla* genes based on their putative peptide length. The distribution of *bla* alleles was highly correlated with *S. saprophyticus* lineages and clusters (χ^2^ test, both *P*-values are 0.0005; Fig. 3A). Isolates with allele 6 and the truncated *bla* (T54) did not show β-lactamase activity. The alignment of *bla* alleles highlights the unique mutations in allele 6, A120T and T215D, located in the catalytic domain (47–263) referring to the features of β-lactamase (UniProt-Q49V79_STAS1) protein of *S. saprophyticus* type strain ATCC 15305 (*E*-value is 8.01e-186). The Delta Delta G (DDG) values of these amino acid substitutions were −0.48 to −0.61 and −1.30 to −1.03 at normal urine pH [5.5–7.54 (50)], predicted by I-Mutant (51), suggesting their potential of decreasing protein stability and influencing β-lactamase productions (Fig. 3D; Fig. S3C). We also observed that 10 out of 21 *mecA* genes in our cohort were identified in isolates carrying T54 *bla* (*n* = 38), which were clustered together in the core gene phylogeny (Fig. S3D), indicating a different evolutionary history of these isolates. Furthermore, given the function of *mecA* against cefoxitin and oxacillin (Fig. 2D), we compared the resistances of *bla* alleles in *mecA*^−^ isolates. The resistance rate against cefoxitin or oxacillin varied by the presence of different *bla* alleles (Fig. 3E and F), suggesting their roles in resisting these two β-lactam agents in *S. saprophyticus*. However, we could not only rely on *bla* alleles or β-lactamase production to predict susceptibility phenotypes of cefoxitin or oxacillin (Fig. S3E), and more genomic determinants needed to be discovered to explain and predict the AMR.

### Putative antibiotic resistance determinants were detected in *S. saprophyticus* against β-lactams

To further address the knowledge gap in genomic determinants of cefoxitin and oxacillin resistance, particularly among *mecA*^−^ *S. saprophyticus* (*n* = 254), we utilized computational models to identify genomic correlates of susceptibility phenotypes. All accessory genes present in at least 10 isolates, and 131 unique amino acid substitutions (referred to as “gene alleles” later) across 36 core genes served as candidates for the correlation analysis (Table S3). Analogs of these 36 genes are reported to be essential for β-lactam resistance in other staphylococci, especially *S. aureus* (52, 53), and are involved in encoding PBPs, cell envelope synthesis, stress responses, nucleotide metabolism, or metal homeostasis (Table S4). 105 genes and 15 gene alleles were significantly correlated with cefoxitin resistance (Table S5) detected by MaAsLin2 (54) with a *q*-value threshold for significance as 0.25. The top features anticorrelated or correlated with cefoxitin, respectively, were gene group_1086 (6/198 isolates with this gene resistant to cefoxitin vs 11/56 isolates absent of this gene resistant to cefoxitin, the coefficient is −0.079), *mgrA*_2 (6/198 vs 11/56, −0.079), *pdhD*_2 (6/198 vs 11/56, −0.079)*,* and gene alleles of *prkC* (8/32 vs 9/222, 0.064), *gdpP* (8/37 vs 9/217, 0.061), and *murF* (8/38 vs 9/216, 0.060). On the other hand, 528 genes and 94 gene alleles exhibited significant correlations with oxacillin resistance (Table S6), and 70.0% of these genes encoded hypothetical proteins. The top correlated features with oxacillin resistance were gene alleles of *pbpH* (41/87 vs 145/167, −0.140), *pyrB* (61/111 vs 125/143, −0.125), *ahpF* (50/94 vs 136/160, −0.116), *glmU* (58/105 vs 128/149, −0.117), *glmS* (31/64 vs 155/190, −0.098), and *mrcA* (59/105 vs 127/149, −0.110), and two genes with unknown functions (group_1734: 0.147 and group_2559: −0.132). These novel candidate gene associations with resistance phenotypes necessitate future functional validation studies in *S. saprophyticus*.

**Fig 4:**
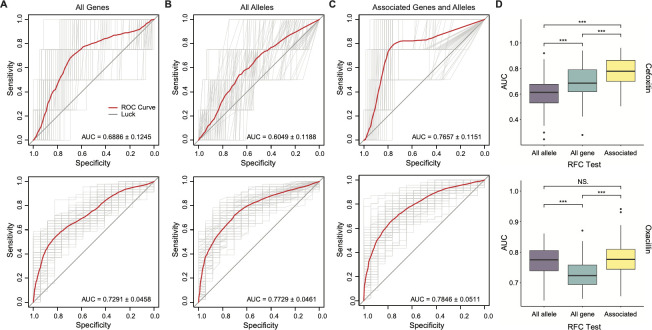
Prediction performances of genotype to phenotype inference for *mecA*^−^ *S. saprophyticus* strains against cefoxitin and oxacillin, tested by RFC. In A–D, the top panel is for cefoxitin, and the bottom panel is for oxacillin. (**A**) ROC curves evaluate the ability to predict the resistance phenotype based on the presence and absence of all accessary genes, presenting in at least 10 isolates. (**B**) ROC curves evaluate the ability to predict the resistance phenotype based on the presence and absence of all gene alleles (present in ≥10 isolates) that have been shown related to methicillin resistance in other staphylococci. (**C**) ROC curves evaluate the ability to predict the resistance phenotype based on the presence and absence of significant AMR-related genes and alleles identified by MaAsLin2. In A–C, the red line represents the mean ROC curves from 100 RPC tests (light gray lines), and the AUC is exhibited for each model. (**D**) Box plots of AUC values from 100 RPC tests of different models in A–C. NS. *P* ≥ 0.05 and ***P < 0.001 as determined by Wilcoxon rank-sum test.

Subsequently, we evaluated the ability to infer susceptibility phenotypes from genotype using a Random Forest Classifier (RFC), trained on the presence or absence of all accessory genes (Fig. 4A), gene alleles (Fig. 4B), or the genes with significant correlations tested above (Fig. 4C). The model with associated genes and gene alleles showed the best prediction performance for both cefoxitin and oxacillin (Fig. 4D): for cefoxitin resistance prediction, its area under the receiver operating characteristic (ROC) curve (AUC; 0.7657 ± 0.1151) was significantly higher than AUCs from the models with all accessory genes (0.6886 ± 0.1245) and gene alleles (0.6049 ± 0.1188) by Wilcoxon rank-sum test (*P*-value is 1.80e-05 and 2.22e-16, respectively); for oxacillin resistance prediction, its AUC (0.7846 ± 0.0511) was significantly higher than the AUC from the model with all accessory genes (0.7291 ± 0.0458) by Wilcoxon rank-sum test (*P*-value is 1.70e-11) but similar with the AUC from the model using gene alleles (0.7729 ± 0.0461, *P*-value is 0.3600).

### Non-β-lactams ARGs previously described in other staphylococci explain most non-β-lactam resistance phenotypes in *S. saprophyticus*

**Fig 5:**
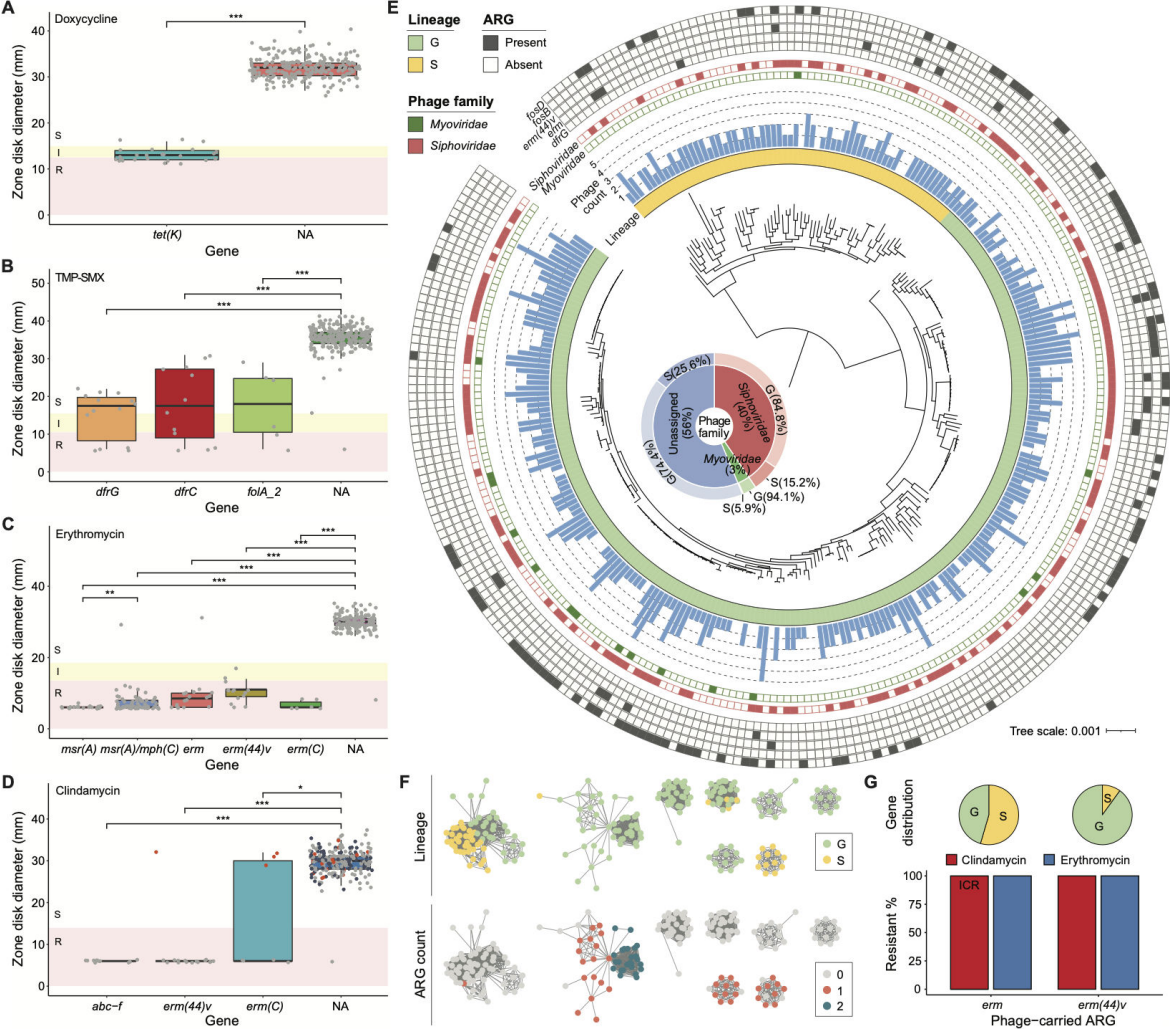
Genes and phage signatures correlated with non-β-lactam resistances in *S. saprophyticus*. (**A–D**) Box plots of zone diameters from disk diffusion tests for doxycycline, TMP-SMX, erythromycin, and clindamycin susceptibility, respectively. The phenotypes are represented by colors: red for resistant, yellow for intermediate, and white for susceptible. Each gray dot denotes an individual isolate carrying a specific ARG. In D, isolates that were tested for ICR by D-test were represented as red or dots for positive or negative results. Few isolates containing different ARGs correlated with the same antibiotics are ignored. NA indicates *S. saprophyticus* with no related ARGs. * *P* < 0.05, **P < 0.01, and ***P < 0.001 as determined by Wilcoxon rank-sum test. (**E**) Annotating phage signatures and phage-carrying ARGs on the core gene phylogenetic tree of *S. saprophyticus*. The scale bar represents the average number of nucleotide substitutions. The innermost ring represents bacterial lineages by color strips. The blue bar graph shows the phage number detected in each *S. saprophyticus* isolate. The distribution of phages from *Siphoviridae* or *Myoviridae* family, as well as the presence/absence of ARGs carried by phages, is visualized as filled and empty symbols. The inset shows the distribution of phage taxonomy in different lineages to their bacterial host belongs. (**F**) Phage populations containing at least 10 phage sequences, defined by MIUVIG-recommended parameters (95% ANI and 85% alignment fraction). Each node represents a phage sequence, and an edge indicates a similarity between its nodes. The phage populations are labeled with their bacterial host lineages (top) and the number of phage-carrying ARGs (bottom). (**G**) Percentage of isolates non-susceptible to clindamycin [ICR for *erm* and constitutive resistance for *erm(44)v*] and erythromycin out of the total number of *S. saprophyticus* carrying *erm* and *erm(44)v* within their phage elements. Distributions of these two genes against lineages are shown at the top.

*S. saprophyticus* isolates in our cohort showed non-susceptibility against four non-β-lactam antibiotics, doxycycline (22/275), TMP-SMX (14/275), erythromycin (129/275), and clindamycin (31/275). The 104 isolates that were erythromycin-resistant and clindamycin-susceptible were tested for inducible clindamycin resistance (ICR) via the disk-diffusion induction test (D-test) (13), and 20/104 isolates showed ICR. We observed significant differences in doxycycline susceptibility phenotypes between isolates with (*n* = 25) and without the *tet(K*) gene (Fig. 5A). Among the *tet(K*)^+^ isolates, three showed susceptible phenotypes against doxycycline. *S. saprophyticus* isolates with *dfrG* (*n* = 14), *dfrC* (*n* = 12), or *folA_2* (*n* = 6) genes showed 35.7% (5/14), 41.7% (5/12), or 50.0% (3/6) non-susceptibility to TMP-SMX (Fig. 5B). For erythromycin AST, isolates with *erm* (*n* = 18), *erm (44)v* (*n* = 14), *erm(C)* (*n* = 6), *msr(A)* (*n* = 20), or *msr(A)/mph(C)* (*n* = 72) genes had 94.4% (17/18), 100.0% (14/14), 100.0% (6/6), 100.0% (20/20), or 98.6% (71/72) non-susceptibility rates (Fig. 5C). Interestingly, *mph(C*) always coexisted with *msr(A*) in a *S. saprophyticus* isolate, a situation that has been described in other CoNS and *S. aureus* (55, 56), and gene sequences of *msr(A*) were different when *mph(C*) was present or absent (Fig. S4A). Lastly, isolates with *abc-f* (*n* = 10), *erm (44)v* (*n* = 14), and *erm(C)* (*n* = 6) genes showed 100.0% (10/10), 92.9% (13/14), or 50.0% (3/6) constitutive resistance rates against clindamycin, which were significantly different from isolates lacking these genes (Fig. 5D). For the four isolates carried *erm (44)v* or *erm(C*), they were not resistant to clindamycin with routine AST but showed ICR by D-test (Fig. 5D red dots). In addition, 17 *S*. *saprophyticus* isolates with *erm* gene did not have constitutive resistance to clindamycin were ICR (57), suggesting the need to detect such resistance by a simple D-test on a routine basis. Accordingly, we observe that typical non-β-lactam ARGs previously reported in staphylococci (Table 1) generally correlated with non-β-lactam phenotypic resistance in *S. saprophyticus*.

**TABLE 1:** Genes associated with non-β-lactam resistance of *S. saprophyticus* in the present work

ARG	Annotation	Isolate number^*a*^	Non-susceptible rate^*b*^	Associated AMR	Reference of the ARG reported in other CoNS
*tet(K*)	Tetracycline efflux MFS^*d*^ transporter	25	88.0%	Doxycycline	Reference (58)
*dfrG*	Trimethoprim-resistant dihydrofolate reductase	14	35.7%	TMP-SMX	Reference (59)
*dfrC*	Trimethoprim-resistant dihydrofolate reductase	12	41.7%	TMP-SMX	References (60, 61)
*folA_2*	Dihydrofolate reductase	6	50.0%	TMP-SMX	References (62, 63)
*msr(A*)	ABC-F type ribosomal protection protein	20	100.0%	Erythromycin	References (64, 65)
*mph(C)^c^*	Mph(C) family macrolide 2'-phosphotransferase	72	98.6%	Erythromycin	References (55, 56)
*erm*	23S ribosomal RNA methyltransferase	18	94.4%	Erythromycin	Reference (66)
*erm (44)v*	23S rRNA (adenine(2058)-N (6))-methyltransferase	14	100.0%, 92.9%, and 100.0%	Erythromycin, Clindamycin, ICR	Reference (67)
*erm(C*)	23S rRNA (adenine(2058)-N (6))-methyltransferase	6	100.0%, 50.0%, and 100.0%	Erythromycin, Clindamycin, ICR	References (56, 68)
*abc-f*	ABC-F type ribosomal protection protein	10	100.0%	Clindamycin	Reference (69)

^*a*^ The number of isolates carrying the ARG.

^*b*^ The ratio of the number of isolates showed phenotypic non-susceptibility to the number of isolates carrying the ARG.

^*c*^ *mph(C*) occurred only in combination with *msr(A*) in the present study.

^*d*^ Major Facilitator Superfamily.

### Erythromycin/clindamycin ARGs are possibly transferred by phage elements

Bacteriophages can act as ARG carriers in various environments (35, 36, 70–72). Phage-encoded ARGs are considered a substantial dissemination threat due to their prolonged persistence, fast replication rate, and potential board host range. Therefore, we assessed the prevalence of phage signatures in our cohort and analyzed their association with phenotypic non-susceptibility among *S. saprophyticus*. By analyzing WGS data, we identified 520 prophage sequences in 91.3% (251/275) isolates (Fig. 5E; Table S7). The average phage number, as well as the proportion of phage-containing isolates, was similar for *S. saprophyticus* from different lineages or clusters (Fig. S5B). After assigning phage taxonomy, we found that 210 of the phages belonged to the *Siphoviridae* family, and 17 were from the *Myoviridae*. The distributions of these two types of phages were different among lineages: 84.8% (178/210) of siphoviruses and 94.1% (16/17) myoviruses were detected in lineage G, whereas 15.2% (32/210) of siphoviruses and 5.9% (1/17) myoviruses were from lineage S (Fig. 5E). We grouped phage sequences with a 95% ANI cutoff and defined each such group as a phage “population.” Isolates from different lineages contained distinctive phage populations (Fig. 5F). This implies either a strain-level specificity of phage infections in *S. saprophyticus* or differential phage environments for G and S isolates.

Next, we detected ARGs in all phage sequences using AMRFinder (73). It found that 20.6% (107/520) of *S. saprophyticus* phages from our cohort carried ARGs, ranging from 1 to 3 (Table S8). Some phage populations contained more ARGs than others (Fig. 5F). These ARGs included *fosD* (*n* = 92), *fosB* (*n* = 7), *erm (44)v* (*n* = 10), *erm* (*n* = 11), and *dfrG* (*n* = 1; Fig. 5E). Since we had performed AST for erythromycin and clindamycin, we then characterized the resistance associations of correlated ARGs within phage elements [i.e., *erm* and *erm (44)v*], which were not found in other parts of the genome. We found *erm* gene was more abundant within S isolates (Fig. 5G; Fig. S5D) and showed high phenotypic non-susceptibility against clindamycin (11/11 ICR) and erythromycin (11/11). Gene *erm (44)v* showed 100.0% constitutive resistance to clindamycin (10/10) and non-susceptibility to erythromycin (10/10; Fig. 5G). In sum, our data suggest an important role for phage elements in encoding and spreading ARGs against erythromycin/clindamycin antibiotics across *S. saprophyticus*.

## DISCUSSION

Here, we present a genomic comparison of 275 human pathogenic *S. saprophyticus* isolates, collected from multicenter healthcare networks. Building from our global phylogenomic characterization of *S. saprophyticus* lineages, we focused on profiling the *S. saprophyticus* antibiotic resistome, including analysis and prediction of genotype-phenotype associations. To test the cefoxitin and oxacillin resistance of our *S. saprophyticus*, we used the disk diffusion method following the procedural guidelines of *Staphylococcus epidermidis* outlined by the CLSI (M100 31st, 2021) (13), given that CLSI is still in the process of defining an optimal surrogate method for *S. saprophyticus*. When comparing susceptibility phenotypes between lineages, G isolates displayed lower phenotypic resistance against oxacillin (146/209 compared to 60/66 among S isolates, *P*-value is 0.0015 by χ^2^ test) but higher resistance against cefoxitin (34/209 compared to 4/66 among S isolates, *P*-value is 0.0435 by χ^2^ test; Fig. 1E). In some *Staphylococcus* species, β-lactam agents, such as oxacillin and cefoxitin, are used as surrogate markers to predict *mecA*-mediated methicillin resistance (74–76). Accordingly, the discrepant burdens of oxacillin and cefoxitin resistance in our cohort prompted us to analyze the occurrence of *mecA* and SCC*mec* elements that transfer *mecA*. Approximately 7.6% (21/275) of our isolates encode *mecA*, which is similar to a previous report from Japan (77). However, the incidence of *mecA* gene in *S. saprophyticus* can be varied across cohorts differing in clinical significance, size, and area (15, 78, 79). We observed that the presence of *mecA* is predictive of oxacillin and cefoxitin resistance for *S. saprophyticus*, but its absence is not predictive of susceptibility. Indeed, 90.3% (186/206) and 44.7% (17/38) of *S. saprophyticus* isolates resistant to oxacillin and cefoxitin, respectively, did not carry *mec* genes. Interestingly, whereas most clinical *S. epidermidis* strains carry *mecA* (80–82), the prevalence of *mecA* in *S. saprophyticus* is much lower. This may indicate that the SCC*mec* mobilization rate or the types of SCC*mec* elements may be different among different staphylococci. A previous study used PCR to characterize the SCC*mec* composition of eight *mecA*^+^ *S. saprophyticus* isolates (83) and found they were all non-typeable according to the current SCC*mec* schemes of *S. aureus* (45) due to the absence of amplification products for hitherto known *ccr* genes. Indeed, we also observed several *mecA*^−^ SCC elements and novel configurations of the *ccr* gene complex in our cohort. One of these non-typable *ccr* complexes, *ccrA1/ccrB3*, was previously reported in *S. saprophyticus* (83) but not other configurations such as *ccrA1/ccrB2*, *ccrA1/ccrB4*, and *ccrA2/ccrB3* (Fig. 2B and C). In contrast, most *mec* complexes found in *S. saprophyticus* were conserved with those in MRSA, implying a similar evolutionary origin. Additionally, two *S*. *saprophyticus* isolates contained *mec* genes adjacent to the mobilization element IS256. This composition has been described in other CoNS, such as *S. epidermidis*, *Staphylococcus haemolyticus*, *Staphylococcus hominis*, *Staphylococcus sciuri*, and *Staphylococcus cohnii* (84, 85) but not in MRSA. IS256 is widespread in the genomes of multiresistant enterococci and staphylococci (86–88). In *S. epidermidis*, IS256 has been recognized as a marker of hospital-acquired MDR and biofilm-forming strains causing opportunistic infections in immunocompromised patients (89–92). In these two *S*. *saprophyticus* strains, we detected the biofilm operon (*ica*) next to the IS265 SCC*mec* that was inverted in the genome compared to other SCC*mec* (Fig. S2B), indicating a unique evolutionary history. Altogether, our findings highlight the diversity of SCC elements among *S. saprophyticus* and motivate further studies on their classification, transmission, and clinical relevance.

Since the *mecA* gene cannot explain all β-lactam resistances in *S. saprophyticus*, we used computational models to identify other potential genomic correlates of the resistance phenotypes, which would be beneficial for future resistance prediction. The results show that cefoxitin and oxacillin-correlated gene features are different, suggesting potentially distinctive resistance mechanisms against these two drugs in *S. saprophyticus*. Among *mecA*^−^ isolates, gene group_1086, *mgrA*, and *pdhD* had positive correlations, and certain gene alleles of *prkC*, *gdpP*, and *murF* had negative correlations with cefoxitin resistance (Table S5). For oxacillin, gene alleles of *pbpH*, *pyrB*, *ahpF*, *glmSU*, and *mrcA* (Table S6) exhibited negative correlations to the resistance phenotype. We observed that several of these genes were involved in cell envelope synthesis and stress response and had been reported to correlate with AMR in other bacterial species. Gene group_1086 is annotated as an alcohol dehydrogenase (ADH) that is widely present among bacteria (93) and mitigates alcohol toxicity (94). A recent study noted that *E. coli adhE* was able to bind with ampicillin and exhibited higher expression levels under ampicillin stress and low intracellular alcohol conditions (95). Next, *mgrA*, a global regulator, has been found to affect several efflux pumps in *S. aureus*, such as *norA* and *norB* for MDR, and *tet38* for tetracycline resistance (96, 97). Gene *pdhD* encodes a membrane protein, dihydrolipoyl dehydrogenase (DLD), which belongs to the oxidoreductase family and is essential for energy metabolism (98). In *Vibrio parahaemolyticus*, DLD levels were upregulated in antimicrobial peptide-resistant clones at both translational and transcriptional levels (99). GdpP is a phosphodiesterase that catalyzes the hydrolysis of intracellular secondary messenger c-di-AMP. *S. aureus gdpP* deletion mutants have been shown to elevate resistance to β-lactams and other cell wall-targeting antimicrobials (100, 101). It also has been shown that mutations of *pyrB*, encoding aspartate carbamoyltransferase, can alter the susceptibility of *P. aeruginosa* to β-lactams (102). Moreover, five types of PBP were detected in our cohort, including PBP2a (*mecA*), PBP 1A (*mrcA*), PBP 2B (*pbpB*), PBP H (*pbpH*), and PBP 1A/1B (*ponA*). Two alleles of *mrcA* and three alleles of *phpH* have been shown to influence oxacillin susceptibilities in *S. saprophyticus* (Table S6). Lastly, in general, single-pass transmembrane proteins with extracellular PBP and serine/threonine kinase-associated (PASTA) domains are important to the cell wall stress response (103, 104). For example, the PASTA kinases of *S. aureus* are essential for β-lactam resistance. However, resistance patterns vary amongst strains, and the mechanism is still understudied (105–107). Genes in the *mur* operon are probably the substrates of *S. aureus* PASTA kinases (108), and *murF* is essential for the optimal expression of methicillin resistance (109). In *S. saprophyticus*, the serine/threonine-protein kinase PrkC is a potential element of the PASTA system. One *prkC* variant and one *murF* variant were correlated with cefoxitin resistance (Tabls S5). In addition, *ahpF* and *glmU* are also identified as potential substrates of PASTA kinases (110, 111). Alkyl hydroperoxide reductase AhpF, protecting cells against reactive oxygen species (112), is important for the tolerance of *E. coli* cells against antibiotics causing DNA damage (113). The GlmSMU pathway responds to produce the Uridine diphosphate-N-acetylglucosamine, an essential peptidoglycan and cell wall teichoic acid precursor (110). We identified alleles of *ahpF* and *glmSU* of *S. saprophyticus* that were anti-correlated with oxacillin resistance (Table S6). In summary, the diverse functions of the above genes indicate the complexity of putative mechanisms of resistance against cefoxitin and oxacillin, which might involve numerous catalytic and metabolic pathways in *S. saprophyticus*. One hypothesis is that genes affecting cell wall construction and stress response can increase bacterial tolerance to survive antibiotic assault. Better resistance prediction performance may be achieved by refining clinical AST breakpoints for *S. saprophyticus* and by including gene expression data from the different observed mutants.

Finally, we characterized phage signatures in *S. saprophyticus*, motivated by the understanding that phages could promote host genetic diversity and niche adaptation by horizontal gene transfer (114–119). In our cohort, the majority of *S. saprophyticus* isolates (91.2%) contained at least one phage element. This high phage genomic prevalence may indicate that they contribute to *S. saprophyticus* fitness in certain environments (37). Earlier work has suggested that phage-carrying ARGs are enriched in the genomes of antibiotic-treated communities (37, 120, 121). Accordingly, we annotated ARGs within phage backbones and evaluated their correlation with phenotypic non-susceptibility since prior reports have suggested they may be non-functional (122). We found that 20.6% (107/520) of *S. saprophyticus* phage sequences contained ARGs, belonging to fosfomycin (*fosB* and *fosD*), macrolide/lincosamide [*erm* and *erm (44)v*], or trimethoprim (*dfrG*) resistance classes. The gene *erm (44*), having around 84% ANI with *erm* in *S. saprophyticus*, was previously reported in a prophage of *Staphylococcus xylosus* and exhibited resistance to erythromycin, together with inducible resistance to clindamycin (66). In contrast, gene *erm (44)v* was originally described in an *S. saprophyticus* isolate and conferred resistance to macrolides and lincosamides (67). We observed that phage-encoding *erm* and *erm (44)v* in our *S. saprophyticus* showed high non-susceptibility against erythromycin/clindamycin. Although neither lincosamides nor macrolides antibiotics were used clinically in the treatment of UTI due to the limited excretion in the urine, we tried to detect their clinical relevance for the *S. saprophyticus* isolates outside the urinary tract. While *S. saprophyticus* is primarily an uropathogen, our cohort included 24.5% (67/275) of non-urinary *S. saprophyticus* isolates; specifically, they were recovered from blood culture (*n* = 46), wounds/tissues/bone (*n* = 15), the respiratory system (*n* = 3), and sterile body fluid (*n* = 3). Surprisingly, the genomic dissimilarity between strains did not correlate with the body site of isolation (Fig. S1F). This may imply that *S. saprophyticus* can transmit and survive in diverse physiological conditions. We then compared the distribution of macrolide/lincosamide phenotypic non-susceptibility and ARGs between isolates from blood, urine, and wound/tissue/bone, and we observed similar distribution in these body sites, besides *msr(A)/mph(C*) gene associated with erythromycin resistance (Fig. S4B and C). Due to the unbalanced sampling, we assumed that we did not have enough statistical power to detect differences across the distribution based on body sites. A focused study of *S. saprophyticus* transmission will require a more balanced sample set in terms of body site of isolation. Further comparative genomics studies should also consider fecal samples from UTI patients, given prior reports of correlations between uropathogen bladder colonization and gastrointestinal colonization (123, 124).

Our study has limitations. Our AST method, disk diffusion, does not generate an minimal inhibitory concentration (MIC) value. However, disk diffusion is a reproducible and standardized CLSI standard method that is widely used in clinical testing and has been well investigated in the setting of *Staphylococcus* spp. relevant to the genotype-phenotype correlation. However, this method relied on manual measurement of the zone of clearance. In addition, only one brand of disk for each of the antibiotics and one brand of Mueller-Hinton agar were evaluated in our study. It is possible that variations of the cation concentration between manufacturers may influence AST results (125).

In summary, we performed a comparative phylogenomic and resistome analysis of a globally diverse collection of 275 human pathogenic *S. saprophyticus* isolates. We compared phenotypic antibiotic susceptibility with potential resistance determinants inferred from current ARG databases and staphylococcal literature. We found that a few documented ARGs [e.g., *tet(K)*, *dfrCG*, *erm*, *erm (44)v*, *erm(C)*, *abc-f*, *msr(A)*, and *msr(A)/mph(C*)] from other staphylococci are associated with phenotypic resistance to doxycycline, TMP-SMX, erythromycin, or clindamycin in *S. saprophyticus* detected in our cohort. In contrast, the genetic antecedents of β-lactam resistance in *S. saprophyticus* are more complicated. Penicillin susceptibility is correlated with *mecA* or *blaZ*. For oxacillin and cefoxitin, the presence of *mecA* is indicative of a resistance phenotype, but the absence of this gene is not predictive of susceptibility to β-lactam antibiotics using current CLSI interpretive criteria. We also identified several genes involved in stress response and cell wall synthesis to be correlated with resistance to these two drugs. Finally, we describe ARG associations with known and novel SCC*mec* configurations as well as phage elements in *S. saprophyticus*, which may serve as intervention or diagnostic targets to limit resistance transmission.

## MATERIALS AND METHODS

### Study cohort

A total of 275 *S*. *saprophyticus* isolates were collected from five medical centers including Washington University School of Medicine in St. Louis (WUSM, *n* = 101), Children’s Hospital Los Angeles (CHLA, *n* = 12), Weill Cornell Medical College (WCMC, *n* = 10), Vanderbilt University Medical Center (VUMC, *n* = 21), University of Alberta Hospital (UA, *n* = 14), and the International Health Management Associates (IHMA, *n* = 117), spanning five continents (South America, *n* = 34; North America, *n* = 172; Europe, *n* = 46; Asia, *n* = 18; Africa, *n* = 5) during 2012–2021 (Table S1). Isolates were recovered from human urine specimens (*n* = 208), blood cultures (*n* = 46), wounds/tissues/bone (*n* = 15), the respiratory system (*n* = 3), and sterile body fluid (*n* = 3). Their purity was evaluated by streaking on blood agar plates (BAPs, Hardy Diagnostics). Microbial identification was confirmed as *S. saprophyticus* using matrix-assisted laser desorption/ionization time-of-flight mass spectrometry with the VITEK MS system (bioMérieux).

### Resistance characterization and Cefinase assay

Susceptibility testing was performed for TMP-SMX, doxycycline, erythromycin, clindamycin, penicillin, and cefoxitin using Hardy Kirby-Bauer Disks (Hardy Diagnostics) and oxacillin using BD BBL disks (Becton, Dickinson and Company). Methods followed the procedural guidelines outlined by the CLSI (documents M02 and M100) (13, 126, 127). Isolates were grown from the frozen stock onto BAPs and subcultured on BAPs and then 3–5 colonies of pure growth were suspended in 0.85% sterile saline at 0.5 McFarland standard. The suspension was used to inoculate a lawn on Mueller-Hinton Agar (MHA, Hardy Diagnostics). After 16–18 hours (24 hours for cefoxitin) incubation, the zone of clearance around the disks was manually measured with a metric ruler. *S. aureus* ATCC 25923 was used as a quality control strain. Detection of β-lactamase production was assessed by nitrocefin-based Cefinase disk test without induced (Hardy Diagnostics).

### Illumina WGS and *de novo* genome assembly

Isolate DNA was extracted manually using the Bacteremia kit (Qiagen) as described previously (128) and was quantified with the Quant-iT PicoGreen double-stranded DNA assay (Thermo Fisher Scientific). 0.5 ng of purified isolate DNA was used as the input to prepare Illumina sequencing libraries with the Nextera kit (Illumina) (129). Libraries were pooled at equal concentrations and sequenced on the NovaSeq 6,000 platform (Illumina) to a minimum depth of 2 million reads per sample (2 × 150 bp). Illumina adapters were removed from demultiplexed reads using Trimmomatic (v0.38) with the following parameters: leading, 10; trailing, 10; sliding window, 4:20; and minimum length, 60 (130). Potential human read contamination was removed using DeconSeq (v0.4.3) (131), and the reads were repaired by BBtools (https://sourceforge.net/projects/bbmap/) with default parameters. Processed reads were *de novo* assembled using Unicycler (v0.4.7) with default settings. Assembly quality was evaluated using BBMap (https://sourceforge.net/projects/bbmap/), QUAST (v4.5) (132), and CheckM (v1.0.13) (133). Assemblies were included for further analysis if (i) they had an average coverage (read depth) ≥40×, (ii) they had a total length within 20.0% range of the reference *S. saprophyticus* strain ASM781411v1 genome size (2.35–3.13 Mbp), (iii) the total number of contigs ≤ 100 and N50 ≥ 10,000, and (iv) with completeness >95.0% and contamination <5.0%.

### Comparative analysis

High-quality assemblies were annotated using Prokka (v1.14.5) with a minimum contig length of 200 bp to identify open reading frames (134). The general feature format (.gff) files outputted by Prokka were used for core gene alignment through Roary (v3.12.0), with default parameters (135). The alignment, composed of 1,646 genes shared by all isolates at a minimum 95.0% identity, was used to generate the maximum likelihood tree with RAxML (v8.2.11) (136). The resulting newick file was visualized in interactive Tree Of Life (iTOL) (137). RhierBAPS (39, 40) was used to identify core gene hierarchical clusters. SNP-sites (v2.4.0) were used to call isolate-specific SNPs against the core gene alignment file created by Roary (138). Whole genome ANI values were determined by FastANI (v1.32) (139) with assembly.fasta files from Unicycler. Next, accessory (non-core) genes identified from Roary are used to calculate the Jaccard distance between isolates through the vegdist function (R vegan package) (140). PCoA was performed on the Jaccard distances using the pcoa function (R ape package) (141).

### ARG and SCC element identification

ARGs were identified by AMRFinder (v3.8.4) (73) using results from Prokka as inputs, including assembled genomes (.fna), predicted genes (.faa), and master annotations (.gff). A presence-absence matrix of all ARGs was generated using MATLAB, with associated metadata displayed as color strips to represent isolate lineage, cluster, and corresponding resistance phenotypes in Fig. 1F. Phage-carrying ARGs were also tested by AMRFinder with 75.0% identity and 50.0% coverage as the threshold for the purpose of identifying the functional ARGs. Gene alignment was performed using Clustal Omega (https://www.ebi.ac.uk/Tools/msa/clustalo/) on extracted sequences at nucleotide or amino acid level. The hierarchical tree based on gene sequence alignment was generated using Jalview (https://www.jalview.org) and visualized with iTOL. All genes of SCC elements were identified and annotated with the online tool SCC*mec*Finder (45) using Roary pan-genome sequence as the input. Hierarchical clustering of *mec* and *ccr* gene contents was performed by the pheatmap function (R pheatmap packages) (142) and labeled with isolate lineage, cluster, and resistance phenotypes by color strips. The alignment of SCC elements was presented by Easyfig (143).

### Prediction accuracy

To evaluate the prediction accuracy links between genotype and phenotype, we applied specific rules related to the presence of ARGs and antibiotic resistance. We assumed that all the ARGs found in the strains were expressed. All *S. saprophyticus* isolates carrying *mecA* were predicted to be resistant to β-lactams, including cefoxitin, oxacillin, and penicillin (Fig. 2D). Isolates carrying *mecA* or *blaZ* were also used to predict penicillin resistance (Fig. S3A). A very major error was defined as inferring susceptibility from genomic data, while the strain was resistant to AST. A major error was defined as inferring resistance from genomic data, while the strain was susceptible by AST. True resistant and true susceptible indicated that the prediction was identical to the AST result.

### Determining phenotype-associated genes and RFC modeling

A presence-absence matrix was built for all accessory genes and 131 unique amino acid substitutions across 36 core genes related to β-lactams reported in other staphylococci (Table S3). Then, this matrix was analyzed by MaAsLin2 (54) to determine the features that were correlated with cefoxitin and oxacillin resistance using the following options: min_prevalence, 0.039 (i.e., present in ≥10 isolates); analysis_method, “LM”; normalization, “CLR”; transform, “None"; others were using the default. RefSeq and UniProtKB accession numbers of the top 8 correlates and the 36 core genes were detected by UniProtBLAST (https://www.uniprot.org/blast) using sequences in pan_genome_reference.fa from Roary (Tables S4 to 6) to check for their annotations and functions. To evaluate the prediction performance from genomic data to resistance phenotype, we conducted a custom machine-learning process employing random forest analysis using the randomForest function (R randomForest package) (144) with default parameters and the following adjustments: ntree = 5,000, proximity = FALSE, importance = TRUE, and mtry = 3. Genes or gene alleles with a prevalence >3.9% (i.e., ≥10 isolates) were included in the analysis. The model was run over 100 iterations of the 75/25 training/testing data set splits. The model performance was measured through the AUC estimator with the prediction and performance functions (R ROCR package) (145). The mean AUC value was reported with 95% confidence intervals. The ROC plot was generated using the predict and roc functions (R pROC package) (146).

### Phage sequence identification and validation

Assemblies from Unicycler were piped through Cenote-Taker 2 to identify putative phage contigs (147) with end features as direct terminal repeats indicating circularity and inverted linear repeats (ITRs) or no features for linear sequences. The linear viral contigs were then binned by VAMB (148) due to the highly fragmented assemblies from short reads, resulting in 1,200 clusters. Contigs in each cluster were concatenated and filtered by length and completeness to remove false positives. Specifically, the length limits were 1,000 nt for the detection of circularity, 4,000 nt for ITRs, and 5,000 nt for other linear sequences. The completeness was computed as a ratio between the length of our phage sequence and the length of matched reference genomes by CheckV (149), and the threshold was set to 10.0%. Phage contigs passed these two filters were then run through VIBRANT with “virome” flag to further remove obvious non-viral sequences (150). As a result, 520 putative viral sequences were identified (Table S7).

### Phage taxonomy and population

Protein sequences created by CheckV were used as input for vConTACT2 with “DIAMOND” and database “ProkaryoticViralRefSeq207-Merged” to assign taxonomy (151). For the “unsigned” ones from vConTACT2, we used the tentative taxonomy from Cenote-Taker 2 inferred using BLASTP against a custom database containing Refseq virus and plasmid sequences from GenBank (147). The final viral taxonomy was determined at the family level and used for further analysis (Table S7). Based on MIUViG recommended parameters (152), phages were grouped into populations if they shared ≥95% nucleotide identity across ≥85% of the genome using BLASTN and a CheckV supporting code, anicalc.py (https://bitbucket.org/berkeleylab/checkv/src/master/). The result was visualized using Cytoscape (https://cytoscape.org).

### Statistical analysis

All statistical tests were performed using the stats (153), vegan (140), ggstatsplot (154), and caret (155) packages in R.

## Data Availability

The adapter removed Illumina reads, and scaffolds of all samples generated in this study have been submitted to the NCBI BioProject database under accession number PRJNA944649.

## SUPPLEMENTAL MATERIAL

Supplemental figures msystems.00697-23-s0001.pdf Fig. S1 to S5.

Table S1 msystems.00697-23-s0002.xlsx Metadata and susceptibility phenotypes of all *S. saprophyticus* isolates in our cohort.

Table S2 msystems.00697-23-s0003.xlsx Matrix of presence and absence of ARGs in each *S. saprophyticus* isolate.

Table S3 msystems.00697-23-s0004.xlsx Matrix of presence and absence of accessory genes and genes alleles for testing cefoxitin and oxacillin resistance determinant candidates.

Table S4 msystems.00697-23-s0005.xlsx Information of 36 core genes whose alleles are used for resistance correlation tests in Table S3.

Table S5 msystems.00697-23-s0006.xlsx Cefoxitin resistance correlated gene and gene alleles in *S. saprophyticus*.

Table S6 msystems.00697-23-s0007.xlsx Oxacillin resistance correlated gene and gene alleles in *S. saprophyticus*.

Table S7 msystems.00697-23-s0008.xlsx Phage sequences identified in our cohort.

Table S8 msystems.00697-23-s0009.xlsx ARGs carried by phages of *S. saprophyticus*.

ASM does not own the copyrights to Supplemental Material that may be linked to, or accessed through, an article. The authors have granted ASM a non-exclusive, world-wide license to publish the Supplemental Material files. Please contact the corresponding author directly for reuse.

## References

[1] Torres Pereira A. 1962. Coagulase-negative strains of staphylococcus possessing antigen 51 as agents of urinary infection. J Clin Pathol 15:252–253. doi:10.1136/jcp.15.3.252 13922034 PMC480392

[2] Ehlers S, Merrill SA. 2022. Staphylococcus saprophyticus. StatPearls, Treasure Island (FL). 29493989

[3] Lee W, Carpenter RJ, Phillips LE, Faro S. 1987. Pyelonephritis and sepsis due to Staphylococcus saprophyticus. J Infect Dis 155:1079–1080. doi:10.1093/infdis/155.5.1079-a 3559281

[4] Matarneh A, Ali GA, Goravey W. 2021. Pyelonephritis-associated Staphylococcus saprophyticus bacteremia in an immunocompetent host: case report and review of the literature. Clin Case Rep 9:e05183. doi:10.1002/ccr3.5183 34917380 PMC8645171

[5] Glimåker M, Granert C, Krook A. 1988. Septicemia caused by Staphylococcus saprophyticus. Scand J Infect Dis 20:347–348. doi:10.3109/00365548809032464 3406676

[6] Singh VR, Raad I. 1990. Fatal Staphylococcus saprophyticus native valve endocarditis in an intravenous drug addict. J Infect Dis 162:783–784. doi:10.1093/infdis/162.3.783 2388008

[7] Hedman P, Ringertz O. 1991. Urinary tract infections caused by Staphylococcus saprophyticus. A matched case control study. J Infect 23:145–153. doi:10.1016/0163-4453(91)92045-7 1753113

[8] Raz R, Colodner R, Kunin CM. 2005. Who are you--Staphylococcus saprophyticus? Clin Infect Dis 40:896–898. doi:10.1086/428353 15736028

[9] Latham RH, Running K, Stamm WE. 1983. Urinary tract infections in young adult women caused by Staphylococcus saprophyticus. JAMA 250:3063–3066. doi:10.1001/jama.1983.03340220031028 6644988

[10] Rupp ME, Soper DE, Archer GL. 1992. Colonization of the female genital tract with Staphylococcus saprophyticus. J Clin Microbiol 30:2975–2979. doi:10.1128/jcm.30.11.2975-2979.1992 1452668 PMC270562

[11] de Sousa VS, da-Silva A de S, Sorenson L, Paschoal RP, Rabello RF, Campana EH, Pinheiro MS, Dos Santos LOF, Martins N, Botelho ACN, Picão RC, Fracalanzza SEL, Riley LW, Sensabaugh G, Moreira BM. 2017. Staphylococcus saprophyticus recovered from humans, food, and recreational waters in Rio de Janeiro, Brazil. Int J Microbiol 2017:4287547. doi:10.1155/2017/4287547 28630628 PMC5463105

[12] de Paiva-Santos W, de Sousa VS, Giambiagi-deMarval M. 2018. Occurrence of virulence-associated genes among Staphylococcus saprophyticus isolated from different sources. Microb Pathog 119:9–11. doi:10.1016/j.micpath.2018.03.054 29604423

[13] CLSI. 2021. M100. Performance standards for antimicrobial susceptibility testing. 31st ed. Clinical and Laboratory Standards Institute, Wayne, PA. 10.1128/JCM.00213-21 PMC8601225 34550809

[14] Ferreira AM, Bonesso MF, Mondelli AL, Camargo CH, Cunha M de L. 2012. Oxacillin resistance and antimicrobial susceptibility profile of Staphylococcus saprophyticus and other staphylococci isolated from patients with urinary tract infection. Chemotherapy 58:482–491. doi:10.1159/000346529 23548376

[15] Hashemzadeh M, Dezfuli AAZ, Nashibi R, Jahangirimehr F, Akbarian ZA. 2021. Study of biofilm formation, structure and antibiotic resistance in Staphylococcus saprophyticus strains causing urinary tract infection in women in Ahvaz, Iran. New Microbes New Infect 39:100831. doi:10.1016/j.nmni.2020.100831 33489239 PMC7807165

[16] Stoesser N, Batty EM, Eyre DW, Morgan M, Wyllie DH, Del Ojo Elias C, Johnson JR, Walker AS, Peto TEA, Crook DW. 2013. Predicting antimicrobial susceptibilities for Escherichia coli and Klebsiella pneumoniae isolates using whole genomic sequence data. J Antimicrob Chemother 68:2234–2244. doi:10.1093/jac/dkt180 23722448 PMC3772739

[17] Tyson GH, McDermott PF, Li C, Chen Y, Tadesse DA, Mukherjee S, Bodeis-Jones S, Kabera C, Gaines SA, Loneragan GH, Edrington TS, Torrence M, Harhay DM, Zhao S. 2015. WGS accurately predicts antimicrobial resistance in Escherichia coli. J Antimicrob Chemother 70:2763–2769. doi:10.1093/jac/dkv186 26142410 PMC11606221

[18] Do Nascimento V, Day MR, Doumith M, Hopkins KL, Woodford N, Godbole G, Jenkins C. 2017. Comparison of phenotypic and WGS-derived antimicrobial resistance profiles of enteroaggregative Escherichia coli isolated from cases of diarrhoeal disease in England, 2015-16. J Antimicrob Chemother 72:3288–3297. doi:10.1093/jac/dkx301 28961934

[19] Ruppé E, Olearo F, Pires D, Baud D, Renzi G, Cherkaoui A, Goldenberger D, Huttner A, François P, Harbarth S, Schrenzel J. 2017. Clonal or not clonal? Investigating hospital outbreaks of KPC-producing Klebsiella pneumoniae with whole-genome sequencing. Clin Microbiol Infect 23:470–475. doi:10.1016/j.cmi.2017.01.015 28143787

[20] Papaventsis D, Casali N, Kontsevaya I, Drobniewski F, Cirillo DM, Nikolayevskyy V. 2017. Whole genome sequencing of Mycobacterium tuberculosis for detection of drug resistance: a systematic review. Clin Microbiol Infect 23:61–68. doi:10.1016/j.cmi.2016.09.008 27665704

[21] Wu X, Tan G, Sha W, Liu H, Yang J, Guo Y, Shen X, Wu Z, Shen H, Yu F. 2022. Use of whole-genome sequencing to predict Mycobacterium tuberculosis complex drug resistance from early positive liquid cultures. Microbiol Spectr 10:e0251621. doi:10.1128/spectrum.02516-21 35311541 PMC9045259

[22] Harris SR, Cole MJ, Spiteri G, Sánchez-Busó L, Golparian D, Jacobsson S, Goater R, Abudahab K, Yeats CA, Bercot B, Borrego MJ, Crowley B, Stefanelli P, Tripodo F, Abad R, Aanensen DM, Unemo M, Euro-GASP study group. 2018. Public health surveillance of multidrug-resistant clones of Neisseria gonorrhoeae in Europe: a genomic survey. Lancet Infect Dis 18:758–768. doi:10.1016/S1473-3099(18)30225-1 29776807 PMC6010626

[23] Yasir M, Karim AM, Malik SK, Bajaffer AA, Azhar EI. 2022. Prediction of antimicrobial minimal inhibitory concentrations for Neisseria gonorrhoeae using machine learning models. Saudi J Biol Sci 29:3687–3693. doi:10.1016/j.sjbs.2022.02.047 35844400 PMC9280306

[24] Jaillard M, van Belkum A, Cady KC, Creely D, Shortridge D, Blanc B, Barbu EM, Dunne WM, Zambardi G, Enright M, Mugnier N, Le Priol C, Schicklin S, Guigon G, Veyrieras J-B. 2017. Correlation between phenotypic antibiotic susceptibility and the resistome in Pseudomonas aeruginosa. Int J Antimicrob Agents 50:210–218. doi:10.1016/j.ijantimicag.2017.02.026 28554735

[25] Khaledi A, Weimann A, Schniederjans M, Asgari E, Kuo T-H, Oliver A, Cabot G, Kola A, Gastmeier P, Hogardt M, Jonas D, Mofrad MR, Bremges A, McHardy AC, Häussler S. 2020. Predicting antimicrobial resistance in Pseudomonas aeruginosa with machine learning-enabled molecular diagnostics. EMBO Mol Med 12:e10264. doi:10.15252/emmm.201910264 32048461 PMC7059009

[26] Sadouki Z, Day MR, Doumith M, Chattaway MA, Dallman TJ, Hopkins KL, Elson R, Woodford N, Godbole G, Jenkins C. 2017. Comparison of phenotypic and WGS-derived antimicrobial resistance profiles of Shigella sonnei isolated from cases of diarrhoeal disease in England and Wales, 2015. J Antimicrob Chemother 72:2496–2502. doi:10.1093/jac/dkx170 28591819

[27] Gordon NC, Price JR, Cole K, Everitt R, Morgan M, Finney J, Kearns AM, Pichon B, Young B, Wilson DJ, Llewelyn MJ, Paul J, Peto TEA, Crook DW, Walker AS, Golubchik T. 2014. Prediction of Staphylococcus aureus antimicrobial resistance by whole-genome sequencing. J Clin Microbiol 52:1182–1191. doi:10.1128/JCM.03117-13 24501024 PMC3993491

[28] Bradley P, Gordon NC, Walker TM, Dunn L, Heys S, Huang B, Earle S, Pankhurst LJ, Anson L, de Cesare M, et al.. 2015. Rapid antibiotic-resistance predictions from genome sequence data for Staphylococcus aureus and Mycobacterium tuberculosis. Nat Commun 6:10063. doi:10.1038/ncomms10063 26686880 PMC4703848

[29] Lee GC, Long SW, Musser JM, Beres SB, Olsen RJ, Dallas SD, Nunez YO, Frei CR. 2015. Comparative whole genome sequencing of community-associated methicillin-resistant Staphylococcus aureus sequence type 8 from primary care clinics in a Texas community. Pharmacotherapy 35:220–228. doi:10.1002/phar.1536 25644979

[30] Lawal OU, Fraqueza MJ, Bouchami O, Worning P, Bartels MD, Gonçalves ML, Paixão P, Gonçalves E, Toscano C, Empel J, Urbaś M, Domínguez MA, Westh H, de Lencastre H, Miragaia M. 2021. Foodborne origin and local and global spread of Staphylococcus saprophyticus causing human urinary tract infections. Emerg Infect Dis 27:880–893. doi:10.3201/eid2703.200852 33622483 PMC7920669

[31] Miragaia M. 2018. Factors contributing to the evolution of mecA-mediated beta-lactam resistance in staphylococci: update and new insights from whole genome sequencing (WGS). Front Microbiol 9:2723. doi:10.3389/fmicb.2018.02723 30483235 PMC6243372

[32] Warsa UC, Nonoyama M, Ida T, Okamoto R, Okubo T, Shimauchi C, Kuga A, Inoue M. 1996. Detection of tet(K) and tet(M) in Staphylococcus aureus of Asian countries by the polymerase chain reaction. J Antibiot (Tokyo) 49:1127–1132. doi:10.7164/antibiotics.49.1127 8982342

[33] Cortes MF, Botelho AM, Almeida LG, Souza RC, de Lima Cunha O, Nicolas MF, Vasconcelos AT, Figueiredo AM. 2018. Community-acquired Methicillin-resistant Staphylococcus aureus from St1 lineage harboring a new Sccmec IV subtype (Sccmec Ivm) containing the tetK gene. Infect Drug Resist 11:2583–2592. doi:10.2147/IDR.S175079 30588041 PMC6299468

[34] Marti E, Variatza E, Balcázar JL. 2014. Bacteriophages as a reservoir of extended-spectrum beta-lactamase and fluoroquinolone resistance genes in the environment. Clin Microbiol Infect 20:456–459. doi:10.1111/1469-0691.12446 24552593

[35] Gómez-Gómez C, Blanco-Picazo P, Brown-Jaque M, Quirós P, Rodríguez-Rubio L, Cerdà-Cuellar M, Muniesa M. 2019. Infectious phage particles packaging antibiotic resistance genes found in meat products and chicken feces. Sci Rep 9:13281. doi:10.1038/s41598-019-49898-0 31527758 PMC6746790

[36] Moon K, Jeon JH, Kang I, Park KS, Lee K, Cha C-J, Lee SH, Cho J-C. 2020. Freshwater viral metagenome reveals novel and functional phage-borne antibiotic resistance genes. Microbiome 8:75. doi:10.1186/s40168-020-00863-4 32482165 PMC7265639

[37] Wendling CC, Refardt D, Hall AR. 2021. Fitness benefits to bacteria of carrying prophages and prophage-encoded antibiotic-resistance genes peak in different environments. Evolution 75:515–528. doi:10.1111/evo.14153 33347602 PMC7986917

[38] Hayati M, Chindelevitch L, Aanensen D, Colijn C. 2022. Deep clustering of bacterial tree images. Philos Trans R Soc Lond B Biol Sci 377:20210231. doi:10.1098/rstb.2021.0231 35989604 PMC9393560

[39] Cheng L, Connor TR, Sirén J, Aanensen DM, Corander J. 2013. Hierarchical and spatially explicit clustering of DNA sequences with BAPS software. Mol Biol Evol 30:1224–1228. doi:10.1093/molbev/mst028 23408797 PMC3670731

[40] Tonkin-Hill G, Lees JA, Bentley SD, Frost SDW, Corander J. 2018. RhierBAPS: an R implementation of the population clustering algorithm hierBAPS. Wellcome Open Res 3:93. doi:10.12688/wellcomeopenres.14694.1 30345380 PMC6178908

[41] Magiorakos AP, Srinivasan A, Carey RB, Carmeli Y, Falagas ME, Giske CG, Harbarth S, Hindler JF, Kahlmeter G, Olsson-Liljequist B, Paterson DL, Rice LB, Stelling J, Struelens MJ, Vatopoulos A, Weber JT, Monnet DL. 2012. Multidrug-resistant, extensively drug-resistant and pandrug-resistant bacteria: an international expert proposal for interim standard definitions for acquired resistance. Clin Microbiol Infect 18:268–281. doi:10.1111/j.1469-0691.2011.03570.x 21793988

[42] Hartman BJ, Tomasz A. 1984. Low-affinity penicillin-binding protein associated with beta-lactam resistance in Staphylococcus aureus. J Bacteriol 158:513–516. doi:10.1128/jb.158.2.513-516.1984 6563036 PMC215458

[43] Ito T, Katayama Y, Hiramatsu K. 1999. Cloning and nucleotide sequence determination of the entire mec DNA of pre-methicillin-resistant Staphylococcus aureus N315. Antimicrob Agents Chemother 43:1449–1458. doi:10.1128/AAC.43.6.1449 10348769 PMC89295

[44] Katayama Y, Ito T, Hiramatsu K. 2000. A new class of genetic element, staphylococcus cassette chromosome mec, encodes methicillin resistance in Staphylococcus aureus. Antimicrob Agents Chemother 44:1549–1555. doi:10.1128/AAC.44.6.1549-1555.2000 10817707 PMC89911

[45] Kaya H, Hasman H, Larsen J, Stegger M, Johannesen TB, Allesøe RL, Lemvigh CK, Aarestrup FM, Lund O, Larsen AR, Limbago BM. 2018. SccmecFinder, a web-based tool for typing of staphylococcal cassette chromosome mec in Staphylococcus aureus using whole-genome sequence data. mSphere 3. doi:10.1128/mSphere.00612-17 PMC5812897 29468193

[46] International Working Group on the Classification of Staphylococcal Cassette Chromosome Elements (IWG-SCC). 2009. Classification of staphylococcal cassette chromosome mec (SCCmec): guidelines for reporting novel SCCmec elements. Antimicrob Agents Chemother 53:4961–4967. doi:10.1128/AAC.00579-09 19721075 PMC2786320

[47] Boundy S, Safo MK, Wang L, Musayev FN, O’Farrell HC, Rife JP, Archer GL. 2013. Characterization of the Staphylococcus aureus rRNA methyltransferase encoded by orfX, the gene containing the staphylococcal chromosome Cassette mec (SCCmec) insertion site. J Biol Chem 288:132–140. doi:10.1074/jbc.M112.385138 23150671 PMC3537007

[48] Monecke S, Jatzwauk L, Müller E, Nitschke H, Pfohl K, Slickers P, Reissig A, Ruppelt-Lorz A, Ehricht R. 2016. Diversity of SCCmec elements in Staphylococcus aureus as observed in South-Eastern Germany. PLoS One 11:e0162654. doi:10.1371/journal.pone.0162654 27648947 PMC5029946

[49] Pratt RF, McLeish MJ. 2010. Structural relationship between the active sites of beta-lactam-recognizing and amidase signature enzymes: convergent evolution?. Biochemistry 49:9688–9697. doi:10.1021/bi1012222 20977193

[50] Bono MJ, Leslie SW, Reygaert WC. 2023. Disclosure: Stephen Leslie declares no relevant financial relationships with ineligible companies, Wanda Reygaert declares no relevant financial relationships with ineligible companies. Urinary tract infection. StatPearls, Treasure Island (FL) ineligible companies.

[51] Capriotti E, Fariselli P, Casadio R. 2005. I-Mutant2.0: predicting stability changes upon mutation from the protein sequence or structure. Nucleic Acids Res 33:W306–W310. doi:10.1093/nar/gki375 15980478 PMC1160136

[52] Pensinger DA, Schaenzer AJ, Sauer JD. 2018. Do shoot the messenger: PASTA kinases as virulence determinants and antibiotic targets. Trends Microbiol 26:56–69. doi:10.1016/j.tim.2017.06.010 28734616 PMC5741517

[53] Kelliher JL, Grunenwald CM, Abrahams RR, Daanen ME, Lew CI, Rose WE, Sauer JD. 2021. PASTA kinase-dependent control of peptidoglycan synthesis via ReoM is required for cell wall stress responses, cytosolic survival, and virulence in Listeria monocytogenes. PLoS Pathog 17:e1009881. doi:10.1371/journal.ppat.1009881 34624065 PMC8528326

[54] Mallick H, Rahnavard A, McIver LJ, Ma S, Zhang Y, Nguyen LH, Tickle TL, Weingart G, Ren B, Schwager EH, Chatterjee S, Thompson KN, Wilkinson JE, Subramanian A, Lu Y, Waldron L, Paulson JN, Franzosa EA, Bravo HC, Huttenhower C. 2021. Multivariable association discovery in population-scale meta-omics studies. PLoS Comput Biol 17:e1009442. doi:10.1371/journal.pcbi.1009442 34784344 PMC8714082

[55] Matsuoka M, Inoue M, Endo Y, Nakajima Y. 2003. Characteristic expression of three genes, msr(A), mph(C) and erm(Y), that confer resistance to macrolide antibiotics on Staphylococcus aureus. FEMS Microbiol Lett 220:287–293. doi:10.1016/S0378-1097(03)00134-4 12670694

[56] Gatermann SG, Koschinski T, Friedrich S. 2007. Distribution and expression of macrolide resistance genes in coagulase-negative staphylococci. Clin Microbiol Infect 13:777–781. doi:10.1111/j.1469-0691.2007.01749.x 17501977

[57] Prabhu K, Rao S, Rao V. 2011. Inducible clindamycin resistance in Staphylococcus aureus isolated from clinical samples. J Lab Physicians 3:25–27. doi:10.4103/0974-2727.78558 21701659 PMC3118052

[58] Trzcinski K, Cooper BS, Hryniewicz W, Dowson CG. 2000. Expression of resistance to tetracyclines in strains of methicillin-resistant Staphylococcus aureus. J Antimicrob Chemother 45:763–770. doi:10.1093/jac/45.6.763 10837427

[59] Nurjadi D, Olalekan AO, Layer F, Shittu AO, Alabi A, Ghebremedhin B, Schaumburg F, Hofmann-Eifler J, Van Genderen PJJ, Caumes E, Fleck R, Mockenhaupt FP, Herrmann M, Kern WV, Abdulla S, Grobusch MP, Kremsner PG, Wolz C, Zanger P. 2014. Emergence of trimethoprim resistance gene dfrG in Staphylococcus aureus causing human infection and colonization in sub-Saharan Africa and its import to Europe. J Antimicrob Chemother 69:2361–2368. doi:10.1093/jac/dku174 24855123

[60] Dale GE, Broger C, Hartman PG, Langen H, Page MG, Then RL, Stüber D. 1995. Characterization of the gene for the chromosomal dihydrofolate reductase (DHFR) of Staphylococcus epidermidis ATCC 14990: the origin of the trimethoprim-resistant S1 DHFR from Staphylococcus aureus?. J Bacteriol 177:2965–2970. doi:10.1128/jb.177.11.2965-2970.1995 7768789 PMC176980

[61] Kyany’a C, Nyasinga J, Matano D, Oundo V, Wacira S, Sang W, Musila L. 2019. Phenotypic and genotypic characterization of clinical Staphylococcus aureus isolates from Kenya. BMC Microbiol 19:245. doi:10.1186/s12866-019-1597-1 31694531 PMC6836327

[62] Sánchez-Osuna M, Cortés P, Llagostera M, Barbé J, Erill I. 2020. Exploration into the origins and mobilization of di-hydrofolate reductase genes and the emergence of clinical resistance to trimethoprim. Microb Genom 6:mgen000440. doi:10.1099/mgen.0.000440 32969787 PMC7725336

[63] Manna MS, Tamer YT, Gaszek I, Poulides N, Ahmed A, Wang X, Toprak FCR, Woodard DR, Koh AY, Williams NS, Borek D, Atilgan AR, Hulleman JD, Atilgan C, Tambar U, Toprak E. 2021. A trimethoprim derivative impedes antibiotic resistance evolution. Nat Commun 12:2949. doi:10.1038/s41467-021-23191-z 34011959 PMC8134463

[64] Ross JI, Eady EA, Cove JH, Baumberg S. 1995. Identification of a chromosomally encoded ABC-transport system with which the staphylococcal erythromycin exporter MsrA may interact. Gene 153:93–98. doi:10.1016/0378-1119(94)00833-e 7883194

[65] Reynolds ED, Cove JH. 2005. Resistance to telithromycin is conferred by msr(A), msrC and msr(D) in Staphylococcus aureus. J Antimicrob Chemother 56:1179–1180. doi:10.1093/jac/dki378 16223938

[66] Wipf JRK, Schwendener S, Perreten V. 2014. The novel macrolide-lincosamide-Streptogramin B resistance gene erm(44) is associated with a prophage in Staphylococcus xylosus. Antimicrob Agents Chemother 58:6133–6138. doi:10.1128/AAC.02949-14 25092709 PMC4187952

[67] Strauss C, Hu Y, Coates A, Perreten V. 2017. A novel erm(44) gene variant from a human Staphylococcus saprophyticus isolate confers resistance to macrolides and lincosamides but not streptogramins. Antimicrob Agents Chemother 61:e01655-16. doi:10.1128/AAC.01655-16 27799208 PMC5192153

[68] Weisblum B. 1995. Erythromycin resistance by ribosome modification. Antimicrob Agents Chemother 39:577–585. doi:10.1128/AAC.39.3.577 7793855 PMC162587

[69] Kadlec K, Schwarz S. 2009. Novel ABC transporter gene, vga(C), located on a multiresistance plasmid from a porcine methicillin-resistant Staphylococcus aureus ST398 strain. Antimicrob Agents Chemother 53:3589–3591. doi:10.1128/AAC.00570-09 19470508 PMC2715595

[70] Colomer-Lluch M, Imamovic L, Jofre J, Muniesa M. 2011. Bacteriophages carrying antibiotic resistance genes in fecal waste from cattle, pigs, and poultry. Antimicrob Agents Chemother 55:4908–4911. doi:10.1128/AAC.00535-11 21807968 PMC3187014

[71] Balcazar JL. 2014. Bacteriophages as vehicles for antibiotic resistance genes in the environment. PLoS Pathog 10:e1004219. doi:10.1371/journal.ppat.1004219 25078987 PMC4117541

[72] Wachino J-I, Jin W, Kimura K, Arakawa Y. 2019. Intercellular transfer of chromosomal antimicrobial resistance genes between Acinetobacter baumannii strains mediated by prophages. Antimicrob Agents Chemother 63:e00334-19. doi:10.1128/AAC.00334-19 31138576 PMC6658751

[73] Feldgarden M, Brover V, Haft DH, Prasad AB, Slotta DJ, Tolstoy I, Tyson GH, Zhao S, Hsu CH, McDermott PF, Tadesse DA, Morales C, Simmons M, Tillman G, Wasilenko J, Folster JP, Klimke W. 2020. Erratum for Feldgarden et al., "validating the AMRfinder tool and resistance gene database by using antimicrobial resistance genotype-phenotype correlations in a collection of isolates". Antimicrob Agents Chemother 64:e00361-20. doi:10.1128/AAC.00361-20 32209564 PMC7179288

[74] Canver MC, Gonzalez MD, Ford BA, Arnold AR, Lawhon SD, Burnham CA, Jenkins SG, Burd EM, Westblade LF. 2019. Improved performance of a rapid immunochromatographic assay for detection of PBP2A in non-Staphylococcus aureus staphylococcal species. J Clin Microbiol 57:e01417-18. doi:10.1128/JCM.01417-18 30651387 PMC6440771

[75] Naccache SN, Callan K, Burnham CA, Wallace MA, Westblade LF, Dien Bard J, Staphylococcus Ad Hoc Working Group of the CLSI Antimicrobial Susceptibility Testing Subcommittee. 2019. Evaluation of oxacillin and cefoxitin disk diffusion and microbroth dilution methods for detecting mecA-mediated Β-lactam resistance in contemporary Staphylococcus epidermidis isolates. J Clin Microbiol 57:e00961-19. doi:10.1128/JCM.00961-19 31462553 PMC6879287

[76] Humphries RM, Magnano P, Burnham CA, Dien Bard J, Dingle TC, Callan K, Westblade LF. 2020. Evaluation of surrogate tests for the presence of mecA-mediated methicillin resistance in Staphylococcus capitis, Staphylococcus haemolyticus, Staphylococcus hominis, and Staphylococcus warneri. J Clin Microbiol 59:e02290-20. doi:10.1128/JCM.02290-20 33115842 PMC7771434

[77] Higashide M, Kuroda M, Ohkawa S, Ohta T. 2006. Evaluation of a cefoxitin disk diffusion test for the detection of mecA-positive methicillin-resistant Staphylococcus saprophyticus. Int J Antimicrob Agents 27:500–504. doi:10.1016/j.ijantimicag.2006.01.009 16697558

[78] Hussain Z, Stoakes L, Massey V, Diagre D, Fitzgerald V, El Sayed S, Lannigan R. 2000. Correlation of oxacillin MIC with mecA gene carriage in coagulase-negative staphylococci. J Clin Microbiol 38:752–754. doi:10.1128/JCM.38.2.752-754.2000 10655380 PMC86195

[79] Xu Z, Shah HN, Misra R, Chen J, Zhang W, Liu Y, Cutler RR, Mkrtchyan HV. 2018. The prevalence, antibiotic resistance and mecA characterization of coagulase negative staphylococci recovered from non-healthcare settings in London, UK. Antimicrob Resist Infect Control 7:73. doi:10.1186/s13756-018-0367-4 29946448 PMC6000976

[80] Rohde H, Kalitzky M, Kröger N, Scherpe S, Horstkotte MA, Knobloch JK-M, Zander AR, Mack D. 2004. Detection of virulence-associated genes not useful for discriminating between invasive and commensal Staphylococcus epidermidis strains from a bone marrow transplant unit. J Clin Microbiol 42:5614–5619. doi:10.1128/JCM.42.12.5614-5619.2004 15583290 PMC535265

[81] Eftekhar F, Raei F. 2011. Correlation of minimum inhibitory concentration breakpoints and methicillin resistance gene carriage in clinical isolates of Staphylococcus epidermidis. Iran J Med Sci 36:213–216. 23359643 PMC3556765

[82] Pourmand MR, Abdossamadi Z, Salari MH, Hosseini M. 2011. Slime layer formation and the prevalence of mecA and aap genes in Staphylococcus epidermidis isolates. J Infect Dev Ctries 5:34–40. doi:10.3855/jidc.984 21330738

[83] Higashide M, Kuroda M, Omura CTN, Kumano M, Ohkawa S, Ichimura S, Ohta T. 2008. Methicillin-resistant Staphylococcus saprophyticus isolates carrying staphylococcal cassette chromosome mec have emerged in urogenital tract infections. Antimicrob Agents Chemother 52:2061–2068. doi:10.1128/AAC.01150-07 18362191 PMC2415764

[84] Couto I, Wu SW, Tomasz A, de Lencastre H. 2003. Development of methicillin resistance in clinical isolates of Staphylococcus sciuri by transcriptional activation of the mecA homologue native to s. J Bacteriol 185:645–653. doi:10.1128/JB.185.2.645-653.2003 12511511 PMC145312

[85] Soumya KR, Sugathan S, Mathew J, Radhakrishnan EK. 2016. Studies on coexistence of mec gene, IS256 and novel sasX gene among human clinical coagulase-negative staphylococci. 3 Biotech 6:233. doi:10.1007/s13205-016-0549-9 PMC5088179 28330305

[86] Loessner I, Dietrich K, Dittrich D, Hacker J, Ziebuhr W. 2002. Transposase-dependent formation of circular IS256 derivatives in Staphylococcus epidermidis and Staphylococcus aureus. J Bacteriol 184:4709–4714. doi:10.1128/JB.184.17.4709-4714.2002 12169594 PMC135277

[87] Vandecraen J, Chandler M, Aertsen A, Van Houdt R. 2017. The impact of insertion sequences on bacterial genome plasticity and adaptability. Crit Rev Microbiol 43:709–730. doi:10.1080/1040841X.2017.1303661 28407717

[88] Kirsch JM, Ely S, Stellfox ME, Hullahalli K, Luong P, Palmer KL, Tyne DV, Duerkop BA. 2022. Targeted IS-element sequencing uncovers transposition dynamics during selective pressure in enterococci. bioRxiv. doi:10.1101/2022.08.24.505136 PMC10266640 37267422

[89] Kozitskaya S, Cho S-H, Dietrich K, Marre R, Naber K, Ziebuhr W. 2004. The bacterial insertion sequence element IS256 occurs preferentially in nosocomial Staphylococcus epidermidis isolates: association with biofilm formation and resistance to aminoglycosides. Infect Immun 72:1210–1215. doi:10.1128/IAI.72.2.1210-1215.2004 14742578 PMC321601

[90] Gu J, Li H, Li M, Vuong C, Otto M, Wen Y, Gao Q. 2005. Bacterial insertion sequence IS256 as a potential molecular marker to discriminate invasive strains from commensal strains of Staphylococcus epidermidis. J Hosp Infect 61:342–348. doi:10.1016/j.jhin.2005.04.017 16242209

[91] Montanaro L, Campoccia D, Pirini V, Ravaioli S, Otto M, Arciola CR. 2007. Antibiotic multiresistance strictly associated with IS256 and ica genes in Staphylococcus epidermidis strains from implant orthopedic infections. J Biomed Mater Res A 83:813–818. doi:10.1002/jbm.a.31399 17559115

[92] Koskela A, Nilsdotter-Augustinsson A, Persson L, Söderquist B. 2009. Prevalence of the ica operon and insertion sequence IS256 among Staphylococcus epidermidis prosthetic joint infection isolates. Eur J Clin Microbiol Infect Dis 28:655–660. doi:10.1007/s10096-008-0664-6 19034541

[93] Gaona-López C, Julián-Sánchez A, Riveros-Rosas H. 2016. Diversity and evolutionary analysis of iron-containing (type-III) alcohol dehydrogenases in eukaryotes. PLoS One 11:e0166851. doi:10.1371/journal.pone.0166851 27893862 PMC5125639

[94] Horinouchi T, Maeda T, Furusawa C. 2018. Understanding and engineering alcohol-tolerant bacteria using OMICS technology. World J Microbiol Biotechnol 34:157. doi:10.1007/s11274-018-2542-4 30341456 PMC6208762

[95] Li L, Yang M, Zhu W, Liu X, Peng X, Li H. 2021. Functionally ampicillin-stressed proteomics reveals that AdhE regulates alcohol metabolism for antibiotic resistance in Escherichia coli. Process Biochem 104:132–141. doi:10.1016/j.procbio.2021.03.017

[96] Truong-Bolduc QC, Zhang X, Hooper DC. 2003. Characterization of NorR protein, a multifunctional regulator of norA expression in Staphylococcus aureus. J Bacteriol 185:3127–3138. doi:10.1128/JB.185.10.3127-3138.2003 12730173 PMC154082

[97] Truong-Bolduc QC, Dunman PM, Strahilevitz J, Projan SJ, Hooper DC. 2005. MgrA is a multiple regulator of two new efflux pumps in Staphylococcus aureus. J Bacteriol 187:2395–2405. doi:10.1128/JB.187.7.2395-2405.2005 15774883 PMC1065235

[98] Dai F, Zhang W, Zhuang Q, Shao Y, Zhao X, Lv Z, Li C. 2019. Dihydrolipoamide dehydrogenase of Vibrio splendidus is involved in adhesion to Apostichopus japonicus. Virulence 10:839–848. doi:10.1080/21505594.2019.1682761 31647357 PMC6816312

[99] Shen CJ, Kuo TY, Lin CC, Chow LP, Chen WJ. 2010. Proteomic identification of membrane proteins regulating antimicrobial peptide resistance in Vibrio parahaemolyticus. J Appl Microbiol 108:1398–1407. doi:10.1111/j.1365-2672.2009.04544.x 19796120

[100] Griffiths JM, O’Neill AJ. 2012. Loss of function of the gdpP protein leads to joint beta-lactam/glycopeptide tolerance in Staphylococcus aureus. Antimicrob Agents Chemother 56:579–581. doi:10.1128/AAC.05148-11 21986827 PMC3256080

[101] Ba X, Kalmar L, Hadjirin NF, Kerschner H, Apfalter P, Morgan FJ, Paterson GK, Girvan SL, Zhou R, Harrison EM, Holmes MA. 2019. Truncation of GdpP mediates beta-lactam resistance in clinical isolates of Staphylococcus aureus. J Antimicrob Chemother 74:1182–1191. doi:10.1093/jac/dkz013 30759229

[102] Alvarez-Ortega C, Wiegand I, Olivares J, Hancock REW, Martínez JL. 2010. Genetic determinants involved in the susceptibility of Pseudomonas aeruginosa to beta-lactam antibiotics. Antimicrob Agents Chemother 54:4159–4167. doi:10.1128/AAC.00257-10 20679510 PMC2944606

[103] Shah IM, Laaberki M-H, Popham DL, Dworkin J. 2008. A eukaryotic-like Ser/Thr kinase signals bacteria to exit dormancy in response to peptidoglycan fragments. Cell 135:486–496. doi:10.1016/j.cell.2008.08.039 18984160 PMC2892110

[104] Fang FC, Frawley ER, Tapscott T, Vázquez-Torres A. 2016. Bacterial stress responses during host infection. Cell Host Microbe 20:133–143. doi:10.1016/j.chom.2016.07.009 27512901 PMC4985009

[105] Beltramini AM, Mukhopadhyay CD, Pancholi V. 2009. Modulation of cell wall structure and antimicrobial susceptibility by a Staphylococcus aureus eukaryote-like serine/threonine kinase and phosphatase. Infect Immun 77:1406–1416. doi:10.1128/IAI.01499-08 19188361 PMC2663143

[106] Donat S, Streker K, Schirmeister T, Rakette S, Stehle T, Liebeke M, Lalk M, Ohlsen K. 2009. Transcriptome and functional analysis of the eukaryotic-type serine/threonine kinase PknB in Staphylococcus aureus. J Bacteriol 191:4056–4069. doi:10.1128/JB.00117-09 19376851 PMC2698490

[107] Tamber S, Schwartzman J, Cheung AL. 2010. Role of PknB kinase in antibiotic resistance and virulence in community-acquired methicillin-resistant Staphylococcus aureus strain USA300. Infect Immun 78:3637–3646. doi:10.1128/IAI.00296-10 20547748 PMC2916262

[108] Liebeke M, Meyer H, Donat S, Ohlsen K, Lalk M. 2010. A metabolomic view of Staphylococcus aureus and its ser/thr kinase and phosphatase deletion mutants: involvement in cell wall biosynthesis. Chem Biol 17:820–830. doi:10.1016/j.chembiol.2010.06.012 20797611

[109] Sobral RG, Ludovice AM, Gardete S, Tabei K, De Lencastre H, Tomasz A. 2003. Normally functioning murF is essential for the optimal expression of methicillin resistance in Staphylococcus aureus. Microb Drug Resist 9:231–241. doi:10.1089/107662903322286436 12959401

[110] Parikh A, Verma SK, Khan S, Prakash B, Nandicoori VK. 2009. PknB-mediated phosphorylation of a novel substrate, N-acetylglucosamine-1-phosphate uridyltransferase, modulates its acetyltransferase activity. J Mol Biol 386:451–464. doi:10.1016/j.jmb.2008.12.031 19121323

[111] Ravikumar V, Shi L, Krug K, Derouiche A, Jers C, Cousin C, Kobir A, Mijakovic I, Macek B. 2014. Quantitative phosphoproteome analysis of Bacillus subtilis reveals novel substrates of the kinase PrkC and phosphatase PrpC. Mol Cell Proteomics 13:1965–1978. doi:10.1074/mcp.M113.035949 24390483 PMC4125730

[112] Chang W, Small DA, Toghrol F, Bentley WE. 2006. Global transcriptome analysis of Staphylococcus aureus response to hydrogen peroxide. J Bacteriol 188:1648–1659. doi:10.1128/JB.188.4.1648-1659.2006 16452450 PMC1367260

[113] Sulaiman JE, Lam H. 2020. Proteomic investigation of tolerant Escherichia coli populations from cyclic antibiotic treatment. J Proteome Res 19:900–913. doi:10.1021/acs.jproteome.9b00687 31920087

[114] Canchaya C, Fournous G, Chibani-Chennoufi S, Dillmann ML, Brüssow H. 2003. Phage as agents of lateral gene transfer. Curr Opin Microbiol 6:417–424. doi:10.1016/s1369-5274(03)00086-9 12941415

[115] Chen J, Quiles-Puchalt N, Chiang YN, Bacigalupe R, Fillol-Salom A, Chee MSJ, Fitzgerald JR, Penadés JR. 2018. Genome hypermobility by lateral transduction. Science 362:207–212. doi:10.1126/science.aat5867 30309949

[116] Frazão N, Sousa A, Lässig M, Gordo I. 2019. Horizontal gene transfer overrides mutation in Escherichia coli colonizing the mammalian gut. Proc Natl Acad Sci U S A 116:17906–17915. doi:10.1073/pnas.1906958116 31431529 PMC6731689

[117] Cook R, Hooton S, Trivedi U, King L, Dodd CER, Hobman JL, Stekel DJ, Jones MA, Millard AD. 2021. Hybrid assembly of an agricultural slurry virome reveals a diverse and stable community with the potential to alter the metabolism and virulence of veterinary pathogens. Microbiome 9:65. doi:10.1186/s40168-021-01010-3 33743832 PMC7981956

[118] Fillol-Salom A, Bacigalupe R, Humphrey S, Chiang YN, Chen J, Penadés JR. 2021. Lateral transduction is inherent to the life cycle of the archetypical Salmonella phage P22. Nat Commun 12:6510. doi:10.1038/s41467-021-26520-4 34751192 PMC8575938

[119] Borodovich T, Shkoporov AN, Ross RP, Hill C. 2022. Phage-mediated horizontal gene transfer and its implications for the human gut microbiome. Gastroenterol Rep (Oxf) 10:goac012. doi:10.1093/gastro/goac012 35425613 PMC9006064

[120] Fancello L, Desnues C, Raoult D, Rolain JM. 2011. Bacteriophages and diffusion of genes encoding antimicrobial resistance in cystic fibrosis sputum microbiota. J Antimicrob Chemother 66:2448–2454. doi:10.1093/jac/dkr315 21816767

[121] Modi SR, Lee HH, Spina CS, Collins JJ. 2013. Antibiotic treatment expands the resistance reservoir and ecological network of the phage metagenome. Nature 499:219–222. doi:10.1038/nature12212 23748443 PMC3710538

[122] Enault F, Briet A, Bouteille L, Roux S, Sullivan MB, Petit M-A. 2017. Phages rarely encode antibiotic resistance genes: a cautionary tale for virome analyses. ISME J 11:237–247. doi:10.1038/ismej.2016.90 27326545 PMC5315482

[123] Thänert R, Reske KA, Hink T, Wallace MA, Wang B, Schwartz DJ, Seiler S, Cass C, Burnham C-A, Dubberke ER, Kwon JH, Dantas G. 2019. Comparative genomics of antibiotic-resistant uropathogens implicates three routes for recurrence of urinary tract infections. mBio 10:e01977-19. doi:10.1128/mBio.01977-19 31455657 PMC6712402

[124] Thänert R, Choi J, Reske KA, Hink T, Thänert A, Wallace MA, Wang B, Seiler S, Cass C, Bost MH, Struttmann EL, Iqbal ZH, Sax SR, Fraser VJ, Baker AW, Foy KR, Williams B, Xu B, Capocci-Tolomeo P, Lautenbach E, Burnham C-AD, Dubberke ER, Kwon JH, Dantas G, CDC Prevention Epicenters Program. 2022. Persisting uropathogenic Escherichia coli lineages show signatures of niche-specific within-host adaptation mediated by mobile genetic elements. Cell Host Microbe 30:1034–1047. doi:10.1016/j.chom.2022.04.008 35545083 PMC10365138

[125] Girardello R, Bispo PJM, Yamanaka TM, Gales AC. 2012. Cation concentration variability of four distinct Mueller-Hinton agar brands influences polymyxin B susceptibility results. J Clin Microbiol 50:2414–2418. doi:10.1128/JCM.06686-11 22553247 PMC3405622

[126] CLSI. 2013. Twenty-third informational supplement, M100-S23. In Performance standards for antimicrobial susceptibility testing. Clinical and Laboratory Standards Institute, Wayne, PA.

[127] CLSI. 2018. M02. Performance standards for antimicrobial susceptibility testing. 13th ed. Clinical and Laboratory Standards Institute, Wayne, PA.

[128] Potter RF, Lainhart W, Twentyman J, Wallace MA, Wang B, Burnham C-A, Rosen DA, Dantas G. 2018. Population structure, antibiotic resistance, and uropathogenicity of Klebsiella variicola. mBio 9:e02481-18. doi:10.1128/mBio.02481-18 30563902 PMC6299229

[129] Baym M, Kryazhimskiy S, Lieberman TD, Chung H, Desai MM, Kishony R. 2015. Inexpensive multiplexed library preparation for megabase-sized genomes. PLoS One 10:e0128036. doi:10.1371/journal.pone.0128036 26000737 PMC4441430

[130] Bolger AM, Lohse M, Usadel B. 2014. Trimmomatic: a flexible trimmer for Illumina sequence data. Bioinformatics 30:2114–2120. doi:10.1093/bioinformatics/btu170 24695404 PMC4103590

[131] Schmieder R, Edwards R, Rodriguez-Valera F. 2011. Fast identification and removal of sequence contamination from genomic and metagenomic datasets. PLoS One 6:e17288. doi:10.1371/journal.pone.0017288 21408061 PMC3052304

[132] Gurevich A, Saveliev V, Vyahhi N, Tesler G. 2013. QUAST: quality assessment tool for genome assemblies. Bioinformatics 29:1072–1075. doi:10.1093/bioinformatics/btt086 23422339 PMC3624806

[133] Parks DH, Imelfort M, Skennerton CT, Hugenholtz P, Tyson GW. 2015. CheckM: assessing the quality of microbial genomes recovered from isolates, single cells, and metagenomes. Genome Res 25:1043–1055. doi:10.1101/gr.186072.114 25977477 PMC4484387

[134] Seemann T. 2014. Prokka: rapid prokaryotic genome annotation. Bioinformatics 30:2068–2069. doi:10.1093/bioinformatics/btu153 24642063

[135] Page AJ, Cummins CA, Hunt M, Wong VK, Reuter S, Holden MTG, Fookes M, Falush D, Keane JA, Parkhill J. 2015. Roary: rapid large-scale prokaryote pan genome analysis. Bioinformatics 31:3691–3693. doi:10.1093/bioinformatics/btv421 26198102 PMC4817141

[136] Stamatakis A. 2014. RAxML version 8: a tool for phylogenetic analysis and post-analysis of large phylogenies. Bioinformatics 30:1312–1313. doi:10.1093/bioinformatics/btu033 24451623 PMC3998144

[137] Letunic I, Bork P. 2019. Interactive Tree Of Life (iTOL) v4: recent updates and new developments. Nucleic Acids Res 47:W256–W259. doi:10.1093/nar/gkz239 30931475 PMC6602468

[138] Page AJ, Taylor B, Delaney AJ, Soares J, Seemann T, Keane JA, Harris SR. 2016. SNP-sites: rapid efficient extraction of SNPs from multi-FASTA alignments. Microb Genom 2:e000056. doi:10.1099/mgen.0.000056 28348851 PMC5320690

[139] Jain C, Rodriguez-R LM, Phillippy AM, Konstantinidis KT, Aluru S. 2018. High throughput ANI analysis of 90K prokaryotic genomes reveals clear species boundaries. Nat Commun 9:5114. doi:10.1038/s41467-018-07641-9 30504855 PMC6269478

[140] Oksanen SG, Blanchet F, Kindt R, Legendre P, Minchin P, O’Hara R, Solymos P, Stevens M, Szoecs E, Wagner H, et al.. 2022. vegan: community ecology package. R package version 2.6-2. https://CRAN.R-project.org/package=vegan, https://CRAN.R-project.org/package=vegan.

[141] Paradis E, Schliep K. 2019. ape 5.0: an environment for modern phylogenetics and evolutionary analyses in R. Bioinformatics 35:526–528. doi:10.1093/bioinformatics/bty633 30016406

[142] Kolde R. 2019. pheatmap: pretty heatmaps, v 1.0.12. https://CRAN.R-project.org/package=pheatmap, https://CRAN.R-project.org/package=pheatmap.

[143] Sullivan MJ, Petty NK, Beatson SA. 2011. Easyfig: a genome comparison visualizer. Bioinformatics 27:1009–1010. doi:10.1093/bioinformatics/btr039 21278367 PMC3065679

[144] Liaw A, Wiener M. 2002. Classification and regression by randomForest. R News 2:18–22.

[145] Sing T, Sander O, Beerenwinkel N, Lengauer T. 2005. ROCR: visualizing classifier performance in R. Bioinformatics 21:3940–3941. doi:10.1093/bioinformatics/bti623 16096348

[146] Robin X, Turck N, Hainard A, Tiberti N, Lisacek F, Sanchez J-C, Müller M. 2011. pROC: an open-source package for R and S+ to analyze and compare ROC curves. BMC Bioinformatics 12:77. doi:10.1186/1471-2105-12-77 21414208 PMC3068975

[147] Tisza MJ, Belford AK, Domínguez-Huerta G, Bolduc B, Buck CB. 2021. Cenote-Taker 2 democratizes virus discovery and sequence annotation. Virus Evol 7:veaa100. doi:10.1093/ve/veaa100 33505708 PMC7816666

[148] Nissen JN, Johansen J, Allesøe RL, Sønderby CK, Armenteros JJA, Grønbech CH, Jensen LJ, Nielsen HB, Petersen TN, Winther O, Rasmussen S. 2021. Improved metagenome binning and assembly using deep variational autoencoders. Nat Biotechnol 39:555–560. doi:10.1038/s41587-020-00777-4 33398153

[149] Nayfach S, Camargo AP, Schulz F, Eloe-Fadrosh E, Roux S, Kyrpides NC. 2021. CheckV assesses the quality and completeness of metagenome-assembled viral genomes. Nat Biotechnol 39:578–585. doi:10.1038/s41587-020-00774-7 33349699 PMC8116208

[150] Kieft K, Zhou Z, Anantharaman K. 2020. VIBRANT: automated recovery, annotation and curation of microbial viruses, and evaluation of viral community function from genomic sequences. Microbiome 8:90. doi:10.1186/s40168-020-00867-0 32522236 PMC7288430

[151] Bin Jang H, Bolduc B, Zablocki O, Kuhn JH, Roux S, Adriaenssens EM, Brister JR, Kropinski AM, Krupovic M, Lavigne R, Turner D, Sullivan MB. 2019. Taxonomic assignment of uncultivated prokaryotic virus genomes is enabled by gene-sharing networks. Nat Biotechnol 37:632–639. doi:10.1038/s41587-019-0100-8 31061483

[152] Roux S, Adriaenssens EM, Dutilh BE, Koonin EV, Kropinski AM, Krupovic M, Kuhn JH, Lavigne R, Brister JR, Varsani A, et al.. 2019. Minimum Information about an Uncultivated Virus Genome (MIUViG). Nat Biotechnol 37:29–37. doi:10.1038/nbt.4306 30556814 PMC6871006

[153] Team RC. 2022. R: a language and environment for statistical computing. R foundation for statistical computing. https://www.R-project.org/, https://www.R-project.org.

[154] Patil I. 2021. Visualizations with statistical details: the {'ggstatsplot'} approach. JOSS 6:3167. doi:10.21105/joss.03167

[155] Kuhn M. 2022. caret: classification and regression training. https://CRAN.R-project.org/package=caret.

