# Supplementary material for: Comparative genomics reveals the correlations of stress response genes and bacteriophages in developing antibiotic resistance of Staphylococcus saprophyticus
Source: mSystems. 2023 Dec 5;8(6):e00697-23. doi: 10.1128/msystems.00697-23 (PMC10734486; doi:10.1128/msystems.00697-23)
Supplement: Supplemental figures — Fig. S1 to S5. [file msystems.00697-23-s0001.pdf]

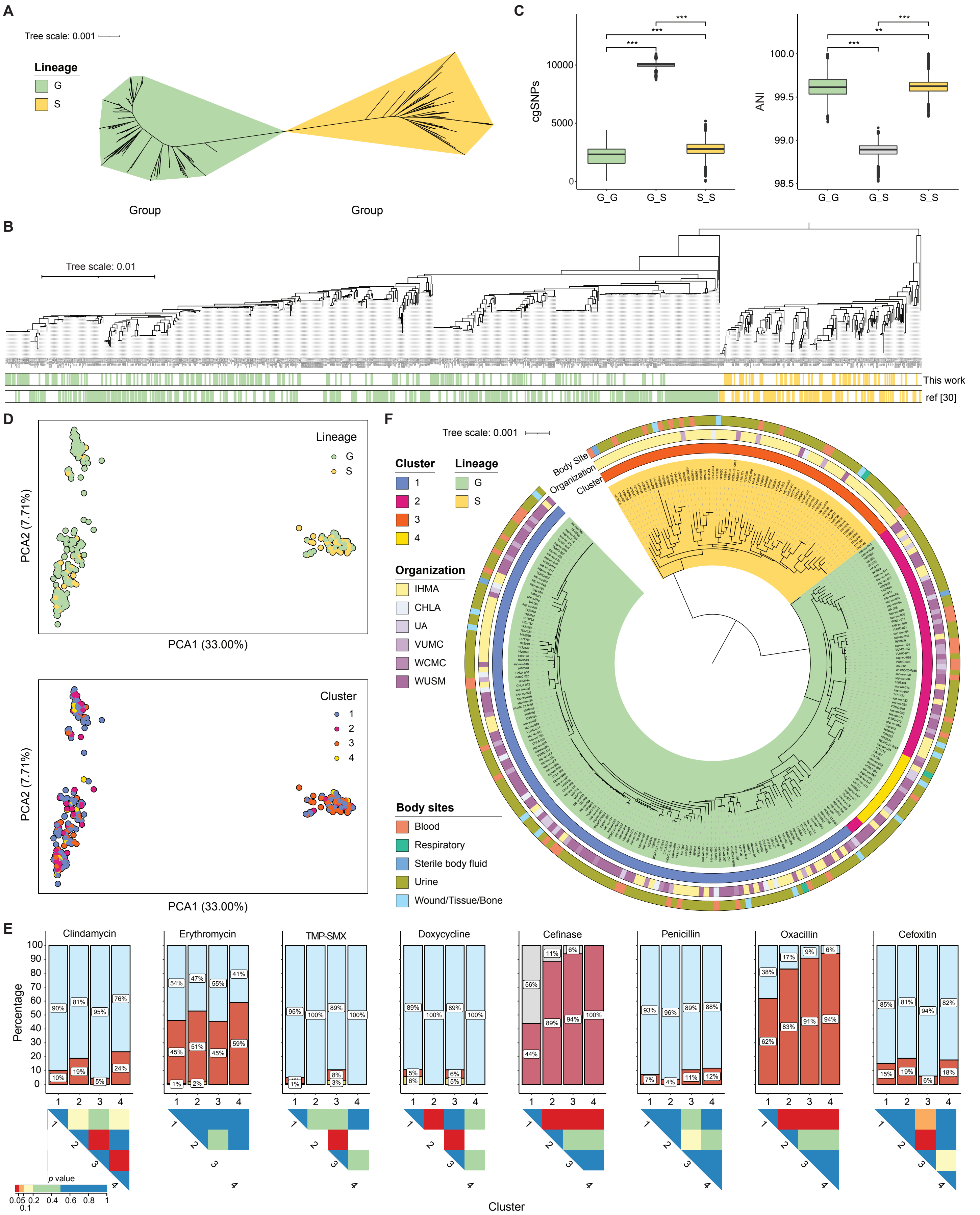

A
Tree scale: 0.001
Lineage
G
S
Group
Group
C
cgSNPs
ANI
G_G
G_S
S_S
***
**
B
Tree scale: 0.01
This work
ref [30]
D
Lineage
PCA2 (7.71%)
PCA1 (33.00%)
Cluster
F
Tree scale: 0.001
Cluster
Lineage
Organization
IHMA
CHLA
UA
VUMC
WCMC
WUSM
Body sites
Blood
Respiratory
Sterile body fluid
Urine
Wound/Tissue/Bone
Body Site
Organization
Cluster
E
Clindamycin
Erythromycin
TMP-SMX
Doxycycline
Cefinase
Penicillin
Oxacillin
Cefoxitin
Percentage
p value
Cluster

Fig. S1 A Unrooted core genome phylogenetic tree showing the water striders shape with a long internal branch separating two distinct sub-populations, G and S. In A and F, the scale bar represents the average number of nucleotide substitutions. **B** Two major lineages of *S. saprophyticus* shown on the core genome phylogenetic tree of 572 published and 275 *S. saprophyticus* genomes from our cohort. Each node represents an isolate. Lineage information of isolates from Lawal et al. and this work are labeled by color strips. **C** Box plots of cgSNPs and whole genome ANI compared between isolates intra and inter lineages. ** $p < 0.01$, *** $p < 0.001$ as determined by Wilcoxon rank-sum test. **D** PcoA ordination of accessory genome similarity as calculated by Jaccard distance. Each dot represents an isolate and axis length represents percent variance. **E** Comparing percentages of isolates resistant to different antibiotics or expressing β-lactamase activity between clusters. Susceptibility phenotypes: resistant, orange; intermediate, yellow; susceptible, light blue. β-lactamase activity: positive, dark blue; negative, light grey. The Chi-square test is used with a significance threshold of 0.05 and associated *p*-values are shown by colors as illustrated on the scale at the bottom left. White signifies NaN. **F** The core genome phylogenetic tree annotated with organizations that collect *S. saprophyticus* isolates of this work and body resources the isolates recovered from by color strips.

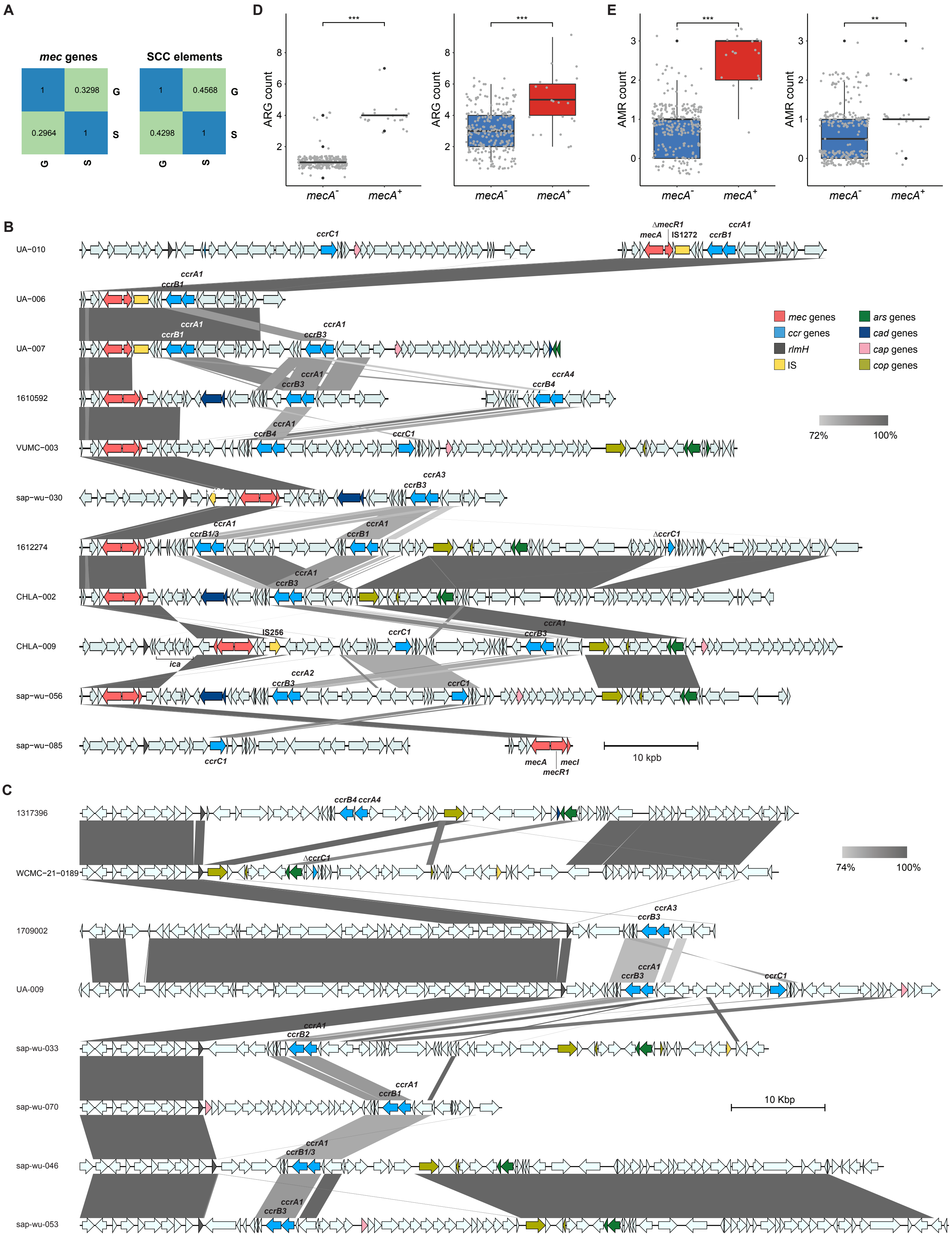

A
mec genes
SCC elements
G
S
1
0.3298
0.2964
0.4568
0.4298
D
E
ARG count
AMR count
mecA-
mecA+
***
**
B
UA-010
UA-006
UA-007
1610592
VUMC-003
sap-wu-030
1612274
CHLA-002
CHLA-009
sap-wu-056
sap-wu-085
mec genes
ccr genes
rlmH
IS
ars genes
cad genes
cap genes
cop genes
72%
100%
10 kpb
ccrA1
ccrB1
ccrC1
ccrB3
ccrA4
ccrB4
ccrA3
ccrB1/3
ΔccrC1
ccrA2
mecA
ΔmecR1
IS1272
IS256
ica
mecI
mecR1
C
1317396
WCMC-21-0189
1709002
UA-009
sap-wu-033
sap-wu-070
sap-wu-046
sap-wu-053
74%
100%
10 Kbp
ccrB2

Fig. S2 A Comparing the prevalence of *mec* genes and SCC elements between lineage G and S. The Chi-square test was used with a significance threshold of 0.05. Associated *p*-values are shown for each comparison group. **B** Alignment of representative SCC*mec* elements with different combinations of *mec* (salmon) and *ccr* (blue) complexes. Isolates containing these sequences are highlighted in red with the same order from top to bottom in Fig. 2B. Arrows represent positions and orientations of open reading frames. Besides *mec* and *ccr* genes, other characteristic genes are color-coded: gene *rlmH* in dark grey, IS in yellow, *ars* genes for arsenic resistance in green, *cad* genes for cadmium resistance in navy, *cap* genes related to capsule synthesis in pink, and cop genes for copper resistance in verdigris. The biofilm operon (*ica*) next to the IS265 SCC*mec* of CHLA-009 (9th sequence from top) is also symbolized. Sequence lengths are presented in scale. Regions between sequences are colored in a gradient of grey, reflecting the percentage of nucleotide identity ranging from 72% to 100%, as illustrated on the scale at the bottom right. **C** Alignment of representative SCC elements with different *ccr* gene complexes in blue. Isolates containing these sequences are highlighted in red Fig. 2C. Arrows symbolize positions and orientations of open reading frames. Certain characteristic genes are color-coded: gene *rlmH* in dark grey, IS in yellow, *ars* genes for arsenic resistance in green, *cad* genes for cadmium resistance in navy, *cap* genes related to capsule synthesis in pink, and cop genes for copper resistance in verdigris. Sequence lengths are presented in scale. Regions between sequences are colored in a gradient of grey, reflecting the percentage of nucleotide identity ranging from 74% to 100%, as illustrated on the scale on the right. **D** Box plots of the numbers of β-lactam (left) and non-β-lactam resistance genes (right) comparing between *mecA*$^-$ and *mecA*$^+$ isolates. **E** Box plots of the numbers of antibiotic reagents each isolate resistant to tested in our work, separated as β-lactam (left) and non-β-lactam resistance phenotype (right), comparing between *mecA*$^-$ and *mecA*$^+$ isolates. ** $p < 0.01$, *** $p < 0.001$ as determined by Wilcoxon rank-sum test.

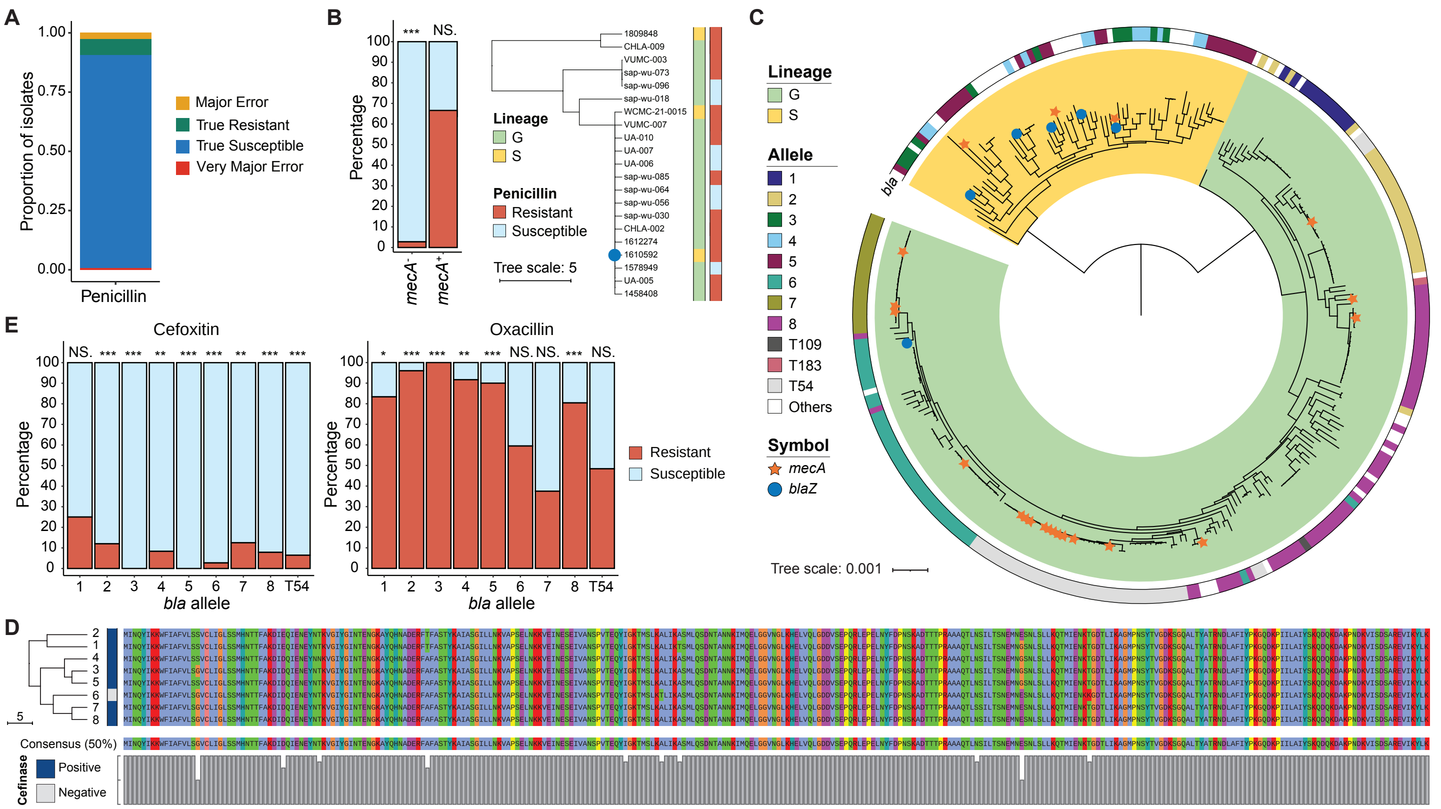

Fig. S3 A Prediction accuracy of genotype to phenotype inference for all *S. saprophyticus* strains of the study. It uses *mecA* and *blaZ* genes as markers to predict the resistance against penicillin. **B** Left: Percentage of isolates resistant to penicillin out of the total number of *mecA*$^-$ and *mecA*$^+$ *S. saprophyticus*. Resistant, orange; susceptible, light blue. Right: Hierarchical tree of 21 *mecA* gene sequences based on AAI. Lineage and penicillin susceptibility phenotype is indicated by color strips. The presence *blaZ* gene in the same isolate is symbolized by the blue circle at the tip of the branch. **C** Annotating the core genome phylogenetic tree with *bla* alleles by color strips. Lineages are indicated with color ranges covering the complete clade branches. The presence of *mecA* and *blaZ* genes is symbolized by the orange star and blue circle respectively at the tip of the branch. The scale bar represents the average number of nucleotide substitutions. **D** Hierarchical tree of *bla* gene alleles 1-8 with the whole-sequence alignment of amino acid sequences (See also Fig. 3D). β-lactamase activity is indicated by color strips. The consensus *bla* sequence shows the amino acid that is found in greater than or equal to 50% of alignments, which is presented with the grey bar graph at the bottom. In B and D, the scale bar represents the average number of amino acid substitutions. **E** Percentage of isolates resistant to cefoxitin and oxacillin out of the total number of *mecA*$^-$ *S. saprophyticus* with specific *bla* alleles. In B and E, the Chi-square test is used with a significance threshold of 0.05. NS. $p \geq 0.05$, * $p < 0.05$, ** $p < 0.01$, *** $p < 0.001$.

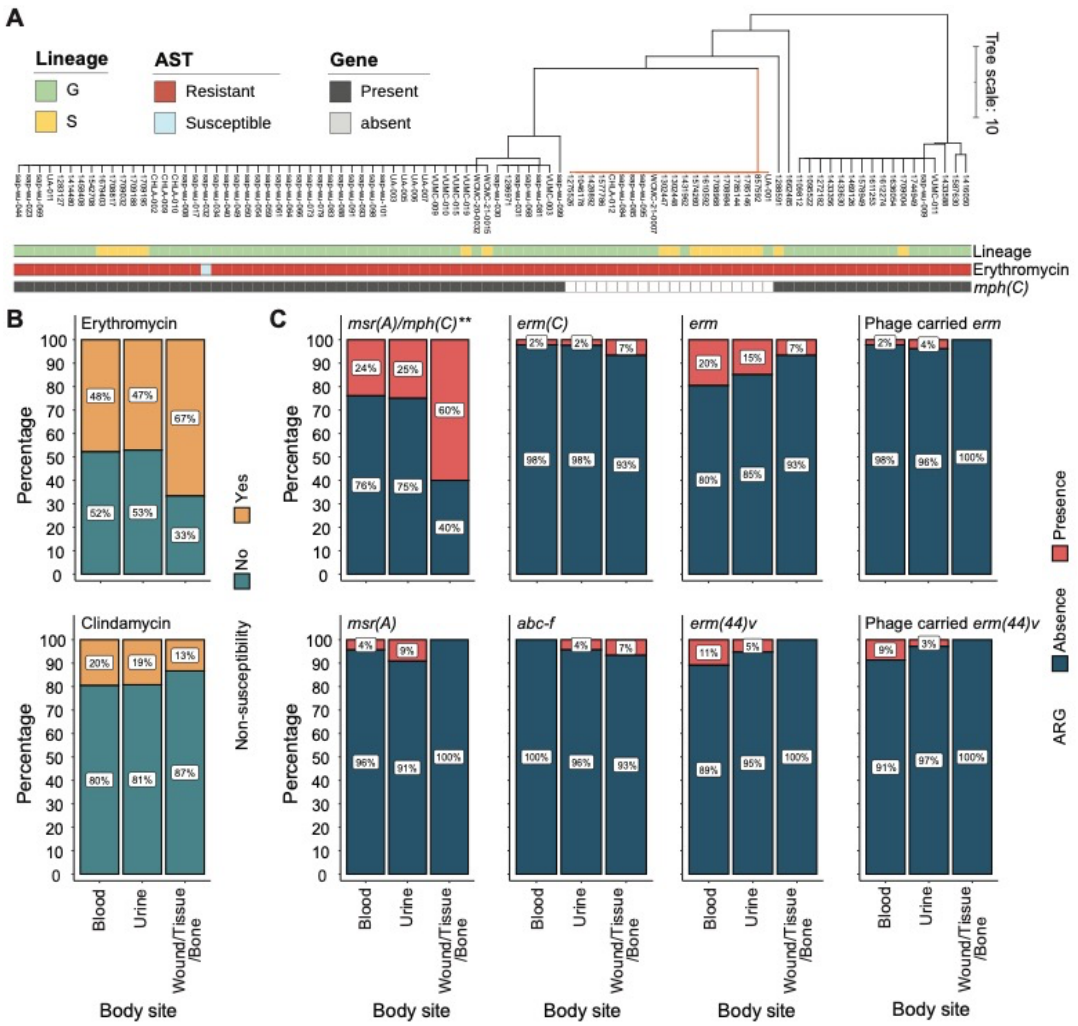

Fig. S4 A Hierarchical tree of *msr(A)* gene sequences based on AAI. The presence/absence of *mph(C)* in the same isolate is visualized as filled and empty symbols. Orange branches highlight the *S. saprophyticus* strains absent of *mph(C)*, where *mrs(A)* has a distinct amino acid sequence. Lineage and antibiotic susceptibility phenotype are indicated by color strips, and the scale bar represents the average number of amino acid substitutions. **B** Comparing percentages of isolates that had non-susceptibility to erythromycin and clindamycin based on body sites. **C** Comparing percentages of isolates carried ARGs related to erythromycin and clindamycin non-susceptibility based on body sites.

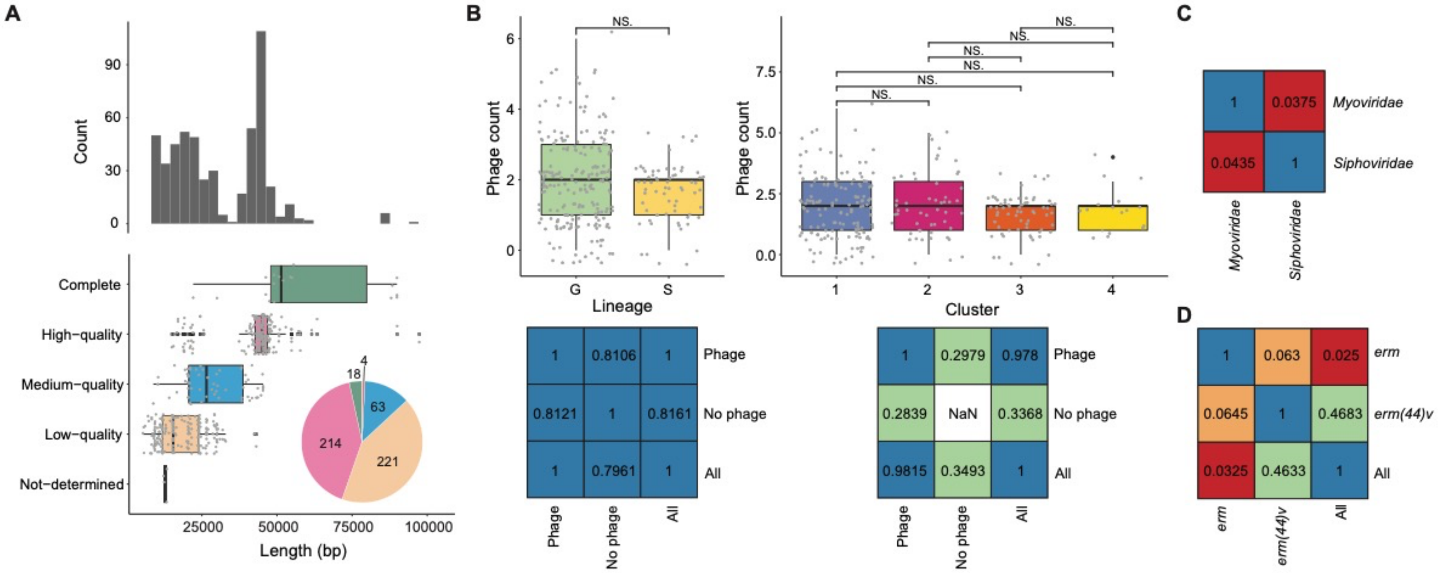

Fig. S5 A Phage sequences identified in our cohort. Top: Distribution of phage length. Bottom: Box plots showing the estimated completeness of phage sequences as a function of their lengths, tested by CheckV. Only the size of the predicted viral region was considered. Compared to the reference phage genome: complete and high quality, >90% completeness; medium quality, 50–90% completeness; low quality, 0–50% completeness; and undetermined quality, no completeness estimates available. The inset exhibits the distribution of completeness labeled with phage count of each category. **B** Top: Box plots of phage numbers comparing between bacterial host lineages (left) and clusters (right). NS. $p \geq 0.05$ as determined by Wilcoxon rank-sum test. Bottom: Comparing the prevalence of phage between lineages (left) and clusters (right) using Chi-square test. **C** Comparing the distribution of phages belonging to *Siphoviridae* and *Myoviridae* families between lineages using Chi-square test. **D** Comparing the prevalence of phage-carrying *erm* and *erm(44)v* between lineages using Chi-square test. In B-D, the Chi-square test is used with a significance threshold of 0.05 and associated *p*-values are shown for each comparison group.
